# Analytical Review on Eccentric Axial Compression Behavior of Short and Slender Circular RC Columns Strengthened Using CFRP

**DOI:** 10.3390/polym13162763

**Published:** 2021-08-17

**Authors:** Muhammad Abid, Haytham F. Isleem, Muhammad Kamal Shah, Shayan Zeb

**Affiliations:** 1 College of Aerospace and Civil Engineering, Harbin Engineering University, Harbin 150001, China; abidkhg@hrbeu.edu.cn (M.A.); mrkamalshah@outlook.com (M.K.S.); shayanzeb62@outlook.com (S.Z.); 2 Department of Civil Engineering, Tsinghua University, Beijing 100084, China

**Keywords:** stress–strain model, slender, circular columns, reinforced concrete, FRP strengthening, axial–flexural interaction

## Abstract

Although reinforced concrete (RC) columns subjected to combined axial compression and flexural loads (i.e., eccentric load) are the most common structural members used in practice, research on FRP-confined circular RC columns subjected to eccentric axial compression has been very limited. More specifically, the available eccentric-loading models were mainly based on existing concentric stress–strain models of FRP-confined unreinforced concrete columns of small scale. The strength and ductility of FRP-strengthened slender circular RC columns predicted using these models showed significant errors. In light of such demand to date, this paper presents a stress–strain model for FRP-confined circular reinforced concrete (RC) columns under eccentric axial compression. The model is mainly based on observations of tests and results reported in the technical literature, in which 207 results of FRP-confined circular unreinforced and reinforced concrete columns were carefully studied and analyzed. A model for the axial-flexural interaction of FRP-confined concrete is also provided. Based on a full parametric analysis, a simple formula of the slenderness limit for FRP-strengthened RC columns is further provided. The proposed model considers the effects of key parameters such as longitudinal and hoop steel reinforcement, level of FRP hoop confinement, slenderness ratio, presence of longitudinal FRP wraps, and varying eccentricity ratio. The accuracy of the proposed model is finally validated through comparisons made between the predictions and the compiled test results.

## 1. Introduction

The building industry plays a significant role in the development of human history. There are various building materials, such as structural materials, decorative materials, and some special materials, that have significantly contributed to the development of the building industry. Structural materials include metal, bamboo, wood, concrete, stone, cement, brick, plastics, ceramics, glass, and composite materials; decorative materials include various coatings, paints, glass with special effects, etc.; special materials refer to waterproof, fire-retardant, heat insulation, etc (i.e., [1]).

With the development of material science and technology, polymer materials exhibit a potential role in the building industry due to their excellent properties compared with inorganic materials. Building polymers commonly used in the construction industry include polyethylene (PE), polyvinyl chloride (PVC), polymethyl methacrylate (PMMA), polyester resin (PR), polystyrene (PS), polypropylene (PP), phenolic resin (PF), and organic silicon resin (OSR). By adding these polymers into traditional building materials, such as concrete and mortar, polymer-based building materials have great benefits when used in construction engineering. Compared with cement concrete, it provides good mechanical strength, short curing period, high adhesion, wear resistance, weather resistance, waterproof, high insulation performance, etc (i.e., [1]).

For fast construction, high-quality control, less waste, and construction interruption, the construction industry is transforming into prefabrication or modularization (i.e., [2,3]). To realize this, the prefabricated elements or buildings exhibit a high strength-to-weight ratio, ease of application, and lightweight. Fiber-reinforced polymers (FRPs) exhibit all these properties and, thus, have been comprehensively used in the construction industry. These composites consist of two components: fibers and matrix. The main functions of fibers are to carry the load and provide stiffness, strength, thermal stability, and other structural properties to the FRP, whereas the matrix ensures the position and alignment of the fibers, protection from damage during manufacture and manipulation, durability of the composite as well as the protection from different effects from the environment. There are several types of fibers in civil structures: carbon (CFRP), glass (GFRP), aramid (AFRP), or basalt (BFRP) fibers.

Due to their high strength and light weight, corrosion resistance, dimensional stability, low thermal conductivity, no conductivity, electromagnetic transparency, impact resistance, and low lifecycle costs, the FRPs provide excellent weather resistance, high durability, adaptable aesthetic appeal, cost-effective manufacturing processes, and other potential benefits. FRPs have been used to replace the traditional construction materials (i.e., steel rebars). In modern buildings. FRPs also have the potential to strengthen and/or retrofit existing concrete structures and reduce the amount of reinforcement and cementitious materials in concrete (i.e., [4,5,6,7]). 

In recent years, significant research has been carried out on the use of fiber-reinforced polymer (FRP) composite materials for enhancing the axial strength, bond strength between concrete and composite materials, shear, deformation, durability, and thermal resistance of concrete (i.e., [8,9,10,11,12,13,14]). The behavior of FRP-confined concrete under axial compression has been widely studied, and as a result, many stress–strain models have been reported. The majority of these models have concentrated largely on plain (unreinforced) FRP-confined concrete cylinders (i.e., [15,16,17,18,19,20,21,22]). To date, only very limited research on FRP-confined circular RC columns of large size has been conducted (i.e., [23,24]).

In reality, concrete columns are subject to eccentric loads, i.e., combined axial compression and flexural loads due to construction errors and accidental load eccentricities resulting from earthquake loads or vehicular loads. Therefore, there has been continuous research effort on the behavior of FRP-confined concrete columns under eccentric loads (e.g., [25,26,27,28,29,30,31,32]). Most of this research has focused on short FRP-confined circular unreinforced and reinforced concrete columns, where the effect of slenderness is negligible (e.g., Wu and Jiang [32]; Cao et al. [33]). It is now commonly accepted that the compressive strength capacity of short columns increases by external FRP confinement to an extent of from 1.5 to 3 times the ordinary columns (e.g., [34]). However, columns with increasing slenderness ratio do not exhibit such strength enhancement levels (e.g., [35]), and the slenderness effect can prohibit the column from reaching its maximum capacity and it may be failed by instability. Therefore, the slenderness effects on eccentrically loaded FRP-confined circular RC columns have also received attention (e.g., [36,37,38]). A general review of relevant works is as follows.

Among existing tests, FRP-strengthened circular RC columns under varying eccentricities were tested by Al-Nimry and Rabadi [25]. The results indicated significant enhancements in the strength of columns due to external FRP confinement when subjected to eccentric loads. The studies also demonstrated reductions in the effectiveness of confinement resulting from varying eccentric loads. Moreover, compared to the unwrapped columns, using a longitudinal FRP wrapping system provided substantial improvements in their flexural resistance.

Tests under constant eccentricity for unwrapped and wrapped circular RC columns of different slenderness ratios and amounts of internal steel and external FRP strengthening were reported by Al-Nimry and Soman [26]. Test variables were studied: slenderness ratio, internal hoop steel reinforcement ratio, amount of hoop FRP sheets, and the absence/presence of longitudinal FRP sheets. Tests showed that strength and ductility, as well as the deformation capacities of columns, can be effectively increased and that their efficiency reduces with increasing the slenderness ratio. Tests also showed that variations in the amount of hoop steel reinforcement had a significant effect on the ductility and that enhancement is less compared to external FRP confinement. Negligible enhancements in the strength of columns with one FRP layer were encountered upon using an additional FRP sheet longitudinally.

Moreover, Fitzwillian and Bisby [28] and Tao et al. [38] have investigated the behavior of FRP-confined circular RC columns with a slenderness ratio (i.e., *l/D*) up to 20.4. The tests revealed reductions in the effectiveness of confinement by increasing the eccentricity and slenderness levels. Further insight into the tests in [28] revealed longitudinal FRP sheets can be used to enhance the performance of slender FRP strengthened circular columns and allow them to attain higher strengths, similar to equivalent short columns laterally confined with FRP. Longitudinal FRP sheets have been observed to have negligible effects on the strength and deformation capacities of the short columns.

All existing literature is experimental work. In terms of model development, only limited research has been concentrated on modeling the behavior of FRP-confined columns under eccentric compression loading. Among them, El Maaddawy [39] proposed a model for the strength and strain of FRP-confined rectangular RC columns, whereas Cao’s model [40] was for FRP-confined circular specimens. The influenced effectiveness of FRP confinement under different eccentricity-to-section depth and slenderness cases on the confined concrete strength and strain at ultimate is not considered in Cao’s model [40]. However, El Maaddawy’s model [39] shows that the strength and strain are inversely proportional to the eccentricity-to-section depth ratio. A model using the moment area method to determine the lateral mid-height deflection is also proposed by El Maaddawy’s model [39].

To address the issues that have not been considered in Cao’s model [40], Hu et al. [41] provided a numerical compression model to evaluate the efficiency of FRP confinement in square and rectangular RC columns under eccentric loads. In their model, the negative effect of increasing the load eccentricity on the ductility gain was considered, and there was a close agreement between strength results of concentric and eccentric tests. These two significant parameters (eccentricity and slenderness ratios) were only considered in the ductility model.

Moreover, Song et al. [42] provided an analytical formula for the maximum compressive load concerning unconfined columns based on a regression analysis of parametric results. In their study, FRP-confined square RC columns were tested to verify the proposed model. The effects of eccentricity ratio, FRP confinement ratio, and unconfined concrete strength on the enhancement provided by the FRP strengthening ratio were identified.

Additionally, international standards and design guidelines for FRP-strengthening RC structures can be found worldwide. Most of them consider the axial–flexural response of eccentrically loaded columns (e.g., ISIS Canada [43], CSA S806-02 [44], CNR-DT200/04 [45], ACI 440.2R [46]; GB 50608 [47]; Concrete Society [48]); of these, only two (i.e., GB 50608 [47]; Concrete Society [48]) provide design guidelines to evaluate the ultimate load capacity of slender RC columns confined with FRP. However, the equations have been proposed based on existing tests on small-scale column specimens and they have not been validated using a wide range of test parameters, in particular large-sized columns.

Although there are many experimental and/or analytical studies on FRP-confined concrete columns under axial compression loading (e.g., [49,50,51,52,53,54]), most of the models have not yet considered the effects of slenderness and eccentric loading. Based on an analytical evaluation and interpretation of a comprehensive test database of FRP-confined RC circular columns under eccentric loading, several existing shortcomings are carefully addressed. It was found the existing models that were established based on small-sized FRP-confined cylinders under concentric or eccentric load gave inadequate predictions for slender FRP-confined RC columns. Therefore, an acceptable test database from existing tests on FRP-confined circular concrete columns is first built. A design-oriented stress-strain model is then proposed, based on the observations and results of 207 specimens, and the effects of various parameters are well addressed. Comparisons between the model results and the tests indicated more accuracy compared with existing models.

## 2. Research Significance

Most of the existing experimental and analytical investigations have been concerned with the behavior of FRP-confined concrete columns tested under concentric compression; however, there is only limited understanding of the behavior of FRP-confined concrete under eccentric axial compression. Therefore, many of the existing design guidelines for eccentrically loaded FRP-confined RC columns still use models derived from results of axial compression tests on plain concrete cylinders despite introducing new models that can be applied to RC columns under eccentric axial compression (e.g., Lin and Teng [55]; Wang et al. [31]; Al-Nimry and Al-Rabadi [25]; El Maaddawy [39]; Csuka and Kollár [56]). The stress-strain model of Lam and Teng [34] which was adopted in most of these studies was based on a test database of 76 plain concrete cylinders of a height-to-diameter ratio ranging from 2 to 4 (i.e., *D* = 100–200 mm), and unconfined concrete strength ranging from 26.2 MPa to 55.2 MPa.

**Figure 1:**
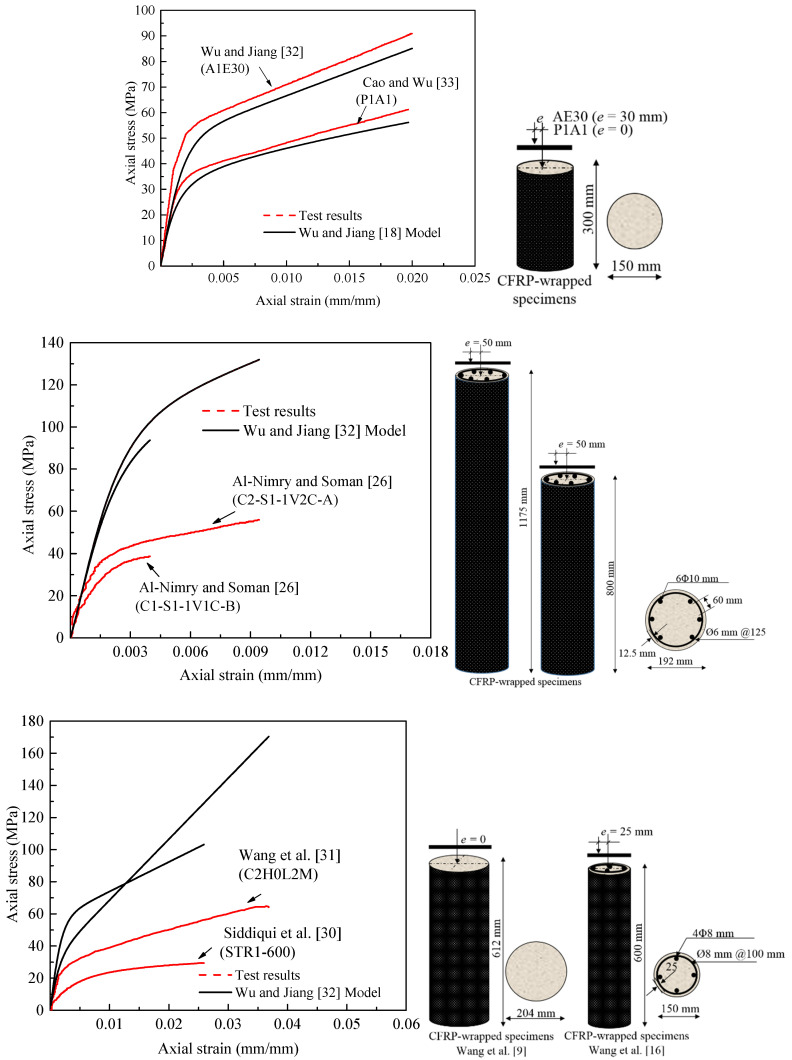
Stress–strain response for FRP-confined columns of small and large scales obtained using Wu and Jiang’s [32] model for FRP-confined concrete cylinders.

Recently, Wu and Jiang [32] have confirmed that the existing stress–strain models derived from concentric loading tests are not suitable for FRP-confined concrete columns under eccentric loading and cannot simulate their response, which has a significant softening trend when the eccentric loading ratio is increased. Based on their tests, an eccentricity-dependent stress-strain model summarized in Table A3 is, therefore, proposed. All specimens used to calibrate their proposed models were 150 mm in diameter and 300 mm in height. The main test parameter was the eccentricity ratio with values of 0, 10, 20, 30, 40, and 50 mm. In sub-section 6.2, the models of Lam and Teng [34], Lin and Jiang [29], and Wu and Jiang [32] have been evaluated. In this discussion, to gain deeper insight into the effect of the Wu and Jiang’s model on stress predictions, Figure 1 shows comparisons between the model predictions with stress-strain test responses of short and slender FRP-confined specimens reported by Cao et al. [33], Wang et al. [31], Siddiqui et al. [30], and Al-Nimry and Soman [26]. It should be noted that when the tested stress-strain curve under concentric loading is not available, the stress–strain model derived by Wei and Wu [15] from concentric loading tests can be used, as in [33]. FRP-confined unreinforced and reinforced columns with different heights and dimensions of cross-sections are provided. The model gives an almost similar global response of FRP-confined concrete cylinders (i.e., *kl/r* = 2) to [32,33]. However, an inspection of the comparisons with larger-sized FRP-confined columns reveals that the model provides very different responses. The response with a higher slenderness ratio has higher errors. Next, a full stress-strain model for slender FRP-confined RC columns is, therefore, developed.

## 3. Experimental Tests

To develop a stress-strain model and also to test the accuracy of the proposed model, a test database of 207 concentrically and eccentrically loaded FRP-confined unreinforced and reinforced concrete columns with different slenderness ratios and material properties (i.e., internal steel ties) was compiled from the literature [23,25,26,27,28,29,30,31,32,57]. The database covers unconfined concrete compressive strength between 21.2 MPa and 59 MPa. All specimens were reinforced with longitudinal and hoop steel bars except those of Jiang et al. [29], Wang et al. [31], Wu and Jiang [32], and few specimens from Wang et al. [23]. All specimens were also strengthened using FRP wraps except for some specimens reported by Al-Nimry and Rabadi [25], Al-Nimry and Soman [26], and Fitzwilliam and Bisby [28], which were reinforced using lateral and longitudinal FRP sheets. To consider the important effects of column slenderness, the column diameter ranges from 150 mm to 305 mm and the height is from 300 to 1200 mm (i.e., *kl/r* = 8–32). Table A1 displays a summary of the tests.

Expressions to predict the peak axial strength were derived by utilizing the results of tests from [23,25,26,27,28,29,30,31,32,57]. The expressions of the corresponding axial strains were mainly based on the results of Wang et al. [23], Al-Nimry and Soman [26], and Fitzwillian and Bisby [28] due to the limited stress-strain responses in the studied literature. Moreover, the lateral deflection model of Section 6 was derived based on results from [26,27,28,29]. To compare the accuracy of the different components of the present model with that of existing models, the models from [34,39,40,41,47,48] were also assessed against the peak strength and strain. Furthermore, the complete stress-strain response was compared previously with the Wu and Jiang [32] model using results of specimens from [26,30,31,32,33], whereas the model of this paper was validated later against the results of the published literature. Finally, the moment interaction diagram was assessed using only the results of Al-Nimry and Rabadi [25] and compared with the existing models [32,34,55].

## 4. Model Development

### 4.1. Effect of Confinement by FRP Wraps

The lateral confinement resulting from the use of FRP wraps to a circular column section is a significant parameter for calculating the peak axial stress and corresponding strain of complete stress–strain response of FRP-confined concrete. The confinement by the FRP hoop wraps is considered using a dimensionless parameter described by Equation (1):

(1) $$\lambda_{f}=\frac{2n_{f}t_{f}E_{f}\varepsilon_{fu}}{Df_{c}^{\prime }}$$

where *E_f_* is the elastic modulus of FRP wraps (MPa); *n_f_* is the number of layers of FRP hoop wraps; *t_f_* is the nominal thickness of an FRP hoop sheet (mm); *D* is the diameter of a circular section (mm); *ɛ_fu_* is the ultimate tensile strain of FRP resulted from flat coupon tests (mm/mm); *f_c_^’^* is the unconfined concrete cylinder strength (MPa).

### 4.2. Effect of Longitudinal FRP Wraps

Tests on the behavior of FRP-wrapped concrete columns have confirmed that using only FRP hoop wraps had a minor effect on the flexural resistance while using longitudinal FRP wraps combined with FRP hoop wraps resulting in significant enhancements in their flexural capacities (e.g., [25,26]). In the study of Siddiqui et al. [30], tests on circular RC columns of different heights (i.e., *l* = 600, 900, 1200 mm) were conducted to study the effect of FRP hoop and longitudinal fibers on the column behavior. It was found in particular that the axial and flexural capacities of slender columns are shared by the longitudinal fibers and that their contributions to the load-carrying capacities of columns with the heights of 900 and 1200 mm are more significant than the shorter ones. The significant efficiency of the longitudinal FRP fibers to slender columns is also reported in Ref. [28], in which the longitudinal FRP sheets do not enhance the performance of concrete cylinders, since these members experience compressive material failure rather than flexural failure. To account for the effect of the longitudinal fibers, the following parameter is introduced (Equation (2)):

(2) $$\lambda_{f,v}=\frac{n_{f,v}E_{f}\varepsilon_{fu}t_{f}}{Df_{c}^{\prime }}$$

where *n_f,v_* is the number of longitudinal layers of FRP sheets.

### 4.3. Effect of Internal Steel Reinforcement r

**Figure 2:**
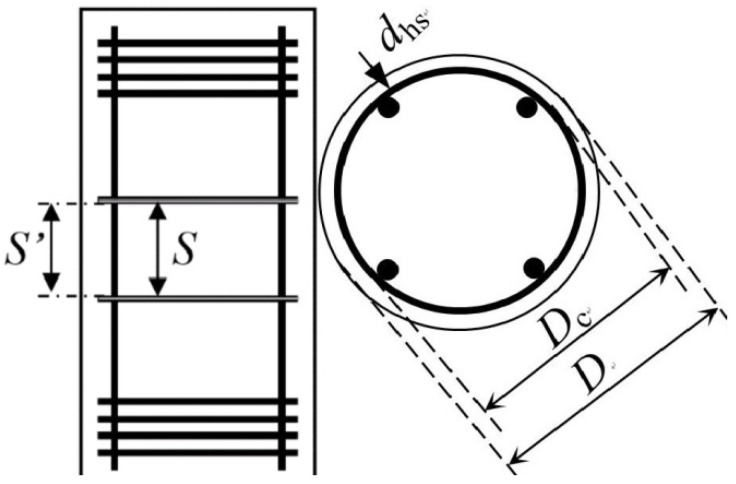
Parameters of *λ_hs_* for columns with internal steel confinement.

Tests on FRP-confined RC columns have revealed a contribution made by the internal hoop reinforcement to the peak strength and strain enhancements (e.g., [23,24,26,58,59,60]), and this contribution is found to be influenced by the amount of FRP wrap, column section size, and slenderness ratio. For example, the effect of internal hoop steel confinement is found to be minimal for columns with an adequate amount of FRP confinement (e.g., Wang et al. [23]). In their study, it has been also found that the effectiveness of FRP reduces as the section size is increased. Among the existing FRP confinement models under eccentric loading as presented in Table A2 and Table A3, one can find the model of Hu et al. [41] that only addresses the effect of the varying slenderness ratios on the effectiveness of FRP confinement. However, the effect of steel confinement is neglected. Therefore, two dimensionless parameters that are relative to the compressive strength of unconfined concrete to consider the effects of steel confinement (*λ_hs_*) and the longitudinal reinforcing steel bars (*λ_vs_*) are provided as:

(3) $$\lambda_{hs}=\frac{\rho_{hs}f_{yh}k_{v}}{f_{c}^{\prime }}$$

(4) $$\lambda_{vs}=\frac{\rho_{vs}f_{yl}}{f_{c}^{\prime }}$$

(5) $$\rho_{hs}=\frac{\pi d_{hs}^{2}}{sD_{c}}$$

where *f_yh_* and *f_yl_* are the yield strengths of the hoop and longitudinal reinforcing steel bars (MPa), respectively; *ρ_hs_* and *ρ_vs_* are the ratios of the hoop and longitudinal steel bars, respectively; *d_hs_* is the diameter of the hoop bar (mm); *D_c_* is the diameter of the concrete core measured to the outside of the hoop bars (mm) (as shown in Figure 2); *s* is the center-to-center vertical spacing of hoop bars (mm). The final coefficient *k_v_* is used herein to quantify the effectiveness of hoop steel confinement in the vertical direction between the hoop reinforcing bars. For concrete columns confined with circular hoop bars, *k_v_* is given in Equation (6) (Mander et al. [60]):

(6) $$k_{v}=\frac{{\left(1-\frac{s^{\prime }}{2D_{c}}\right)}^{2}}{1-\rho_{cc}}$$

where *s*’ is the clear spacing between the hoop steel bars (see Figure 2); *ρ_cc_* is the ratio between the area of longitudinal steel reinforcement to the area of the concrete core, and it can be determined as $\rho cc=\pi {\left(D_{c}/2\right)}^{2}-\rho_{vs}A_{g}$, in which *A_g_* (mm^2^) is the total cross sectional area of the column.

### 4.4. Peak Axial Strength and Strain

The peak strength, *f_cc_^’^*, and strain, *ɛ_cc_*, are two significant requirements for a stress–strain response of FRP-confined concrete. Existing tests on FRP-confined RC columns revealed that *f_cc_^’^* and *ɛ_cc_* are influenced by the level of internal steel confinement, longitudinal and hoop FRP sheets, eccentric load ratio, and slenderness ratio. The ratio of hoop steel reinforcement has a significant effect on the ductility enhancement rather than on the strength enhancement resulting from the FRP confinement [26,61]. For accurate modeling, two expressions (i.e., Equations (7) and (8)) with different ranges of longitudinal and hoop steel reinforcement ratios are provided. Equation (7) was calibrated using all eccentric loading tests, whereas Equation (8) was expanded to consider the concentric tests compiled from Wang et al. [23] and Kaeseberg et al. [57]. The expressions had an averaged correlation coefficient (*R*^2^) of about 93.7% and were based on the analysis of all 207 specimens summarized in Table A1:

(7) $$f_{cc}^{\prime }=\delta_{CRC}f_{c}^{\prime },\left\{ \begin{array}{l} \lambda_{hs}=0-0.09\\ \lambda_{vs}=0-0.19 \end{array}\right.$$

(8) $$f_{cc}^{\prime }=\delta_{CRC}f_{c}^{\prime }+B_{1.1}{\left(\lambda_{hs}+\lambda_{vs}\right)}^{B_{1.2}}+B_{1.3}{\left(\lambda_{f}\right)}^{B_{1.4}},\left\{ \begin{array}{l} \lambda_{hs}=0-0.20\\ \lambda_{vs}=0-0.51 \end{array}\right.$$

(9) $$\delta_{CRC}=\delta_{URC}+\left[B_{2.1}{\left(\lambda_{f}\right)}^{B_{2.2}}+{\left(\lambda_{f,v}\right)}^{B_{2.3}}\right]{\left(1+\frac{e}{D}\right)}^{B_{2.4}}{\left(\frac{l}{D}\right)}^{B_{2.5}}\;(\mathrm{FRP}-\mathrm{confined}\;\mathrm{RC}\;\mathrm{columns})$$

(10) $$\delta_{URC}=\left[B_{3.1}{\left(\frac{l}{D}\right)}^{B_{3.2}}+B_{3.3}\lambda_{vs}\frac{e}{D}\right]{\left(1+\frac{e}{D}\right)}^{B_{3.4}}\;(\mathrm{RC}\;\mathrm{columns})$$

where *l* is column height (mm); *e* is loading eccentricity (mm); *δ_URC_* and *δ_CRC_* (dimensionless coefficients) are strength gains of unwrapped and FRP-wrapped RC columns, respectively. The resulting values of *B*_1.1_, *B*_1.2_, *B*_1.3_, *B*_1.4_ in Equation (8) are 31.53, 1.38, 14.97 and 0.54, respectively. In Equation (9), *B*_2.1_ = 5.264; *B*_2.2_ = 1.295; *B*_2.3_ = 0.643; *B*_2.4_ = −2.733; *B*_2.5_ = −0.614. The coefficients of Equation (10) are obtained as *B*_3.1_ = 1.083; *B*_3.2_ = −0.092; *B*_3.3_ = 4.330; *B*_3.4_ = −2.386.

**Figure 3:**
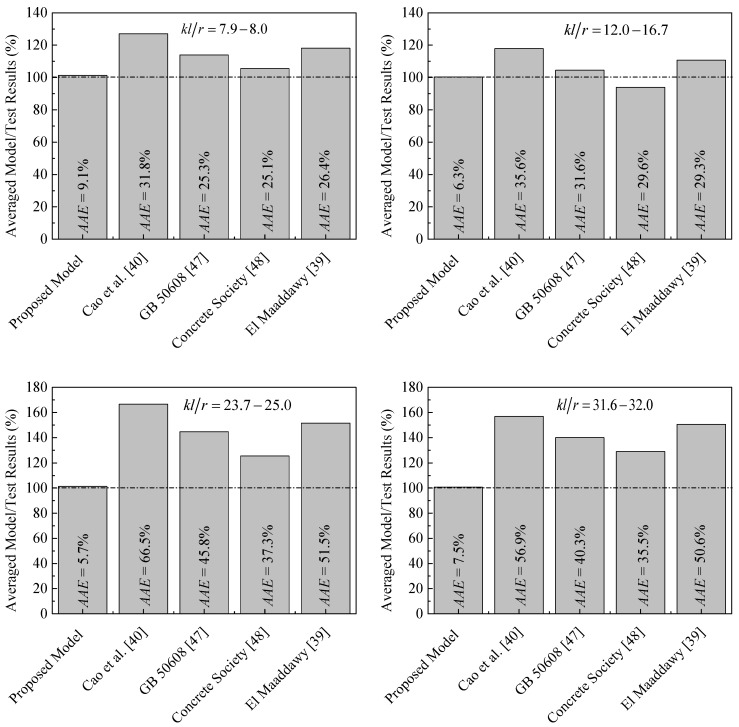
Comparison between proposed and existing peak strength models of FRP-confined concrete under eccentric loading.

The proposed peak strength model is applicable for FRP-confined unreinforced columns, FRP-confined RC columns, and unwrapped RC columns. The accuracy of the proposed and existing expressions is assessed by the average absolute error (*AAE*). Predictions given by the proposed expressions and those of the models [39,40,47,48] are compared with the test results in Figure 3. It is seen that the existing models for tests with slenderness ratios ranging from 7.9 to 17.0 overestimate the results by 11.4% (*AAE* = 29.3). Moreover, the direct use of these models leads to significant errors in predicting the tested peak strength of FRP-confined slender RC columns. In a range of higher slenderness ratios ranging from 23.7 to 32, the experimental results are overestimated by 45.6% (almost increased by four times as compared with the smaller range of slenderness, *kl/r* ≤ 17). The ratio between the analytical results given by the new model and the results equal 101% with an *AAE* value of about 7.2, whereas the ratio between the analytical results from the existing models and the results is equal to 128.5% with an *AAE* value of about 38.7. Finally, it can be concluded that the present model agrees best with the test results.

Similar to the model given in Equation (9), an expression for the peak strain *ɛ_cc_* accounting for the effects of key parameters is provided in Equation (11), in which the correlation coefficients are 91.9 and 88.7% for the first and second parts of the expression, respectively:

(11) $$\frac{\varepsilon_{cc}}{\varepsilon_{co}}=\left\{ \begin{array}{l} \left[1+B_{4.1}{\left(\lambda_{hs}\right)}^{B_{4.2}}+B_{4.3}{\left(\lambda_{f}\right)}^{B_{4.4}}+B_{4.5}\left(\lambda_{f,v}\right)\right]{\left(1+\frac{e}{D}\right)}^{B_{4.6}}{\left(\frac{l}{D}\right)}^{B_{4.7}}(CZ)\\ {\left(\frac{\varepsilon_{cc}}{\varepsilon_{co}}\right)}_{con}-\left[B_{5.1}+B_{5.2}\left(\lambda_{hs}\right)+B_{5.3}{\left(\lambda_{f}\right)}^{B_{5.4}}+B_{5.5}\left(\lambda_{f,v}\right)\right]{\left(1+\frac{e}{D}\right)}^{B_{5.6}}{\left(\frac{l}{D}\right)}^{B_{5.7}}(CZ\;\&\;TZ) \end{array}\right.$$

where *CZ* indicates that the proposed expression can predict the maximum confined strain in the compression zone of the cross-section, whereas *CZ* and *TZ* refer to the ultimate strain in compression and tension section sides, respectively; *ɛ_co_* is the compressive strain corresponding to the peak strength of unconfined concrete and is taken to be 0.002. In the present model, the (*ɛ_cc_*/*ɛ_co_*)_con_ ratio was determined from the concentrically loaded model of Wang et al. [23], as provided in Equations (12)–(14). The values of *B*_4.1_, *B*_4.2_, *B*_4.3_, *B*_4.4_, *B*_4.5_, *B*_4.6_, *B*_4.7_ in Equation (11) are obtained to be 12.23, 0.87, 19.83, 0.66, 3.77, −1.10, 0.11, respectively, whereas in its second part *B*_5.1_ = 0.68, *B*_5.2_ = 3.15, *B*_5.3_ = 7.84, *B*_5.4_ = 0.55, *B*_5.5_ = −0.98, *B*_5.6_ = 0.69, *B*_5.7_ = 0.34.

**Figure 4:**
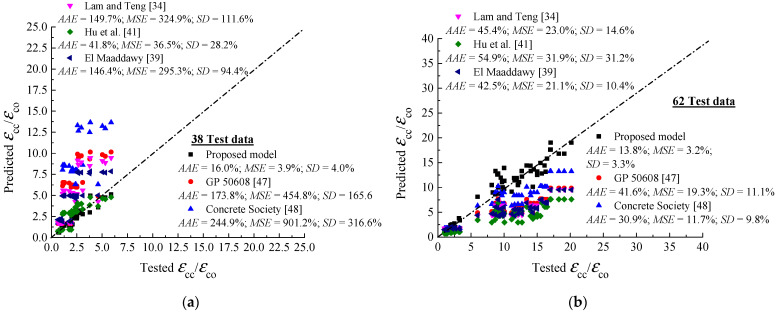
Performance of proposed and existing FRP-confined concrete models of peak strain; (**a**) data recorded on compression and tension zones; (**b**) data recorded on compression zone.

The model proposed for the ultimate strain is also applicable for FRP-confined unreinforced columns, FRP-confined RC columns, and unwrapped RC columns. Predictions given by the proposed Equation (11) and those of the models [34,39,41,47,48] are compared with the tested strains in Figure 4. Among the presented models, the proposed model has the best correlation between the analytical and experimental results. In addition, the error of the proposed model is insignificant when compared with those of the existing models:

(12) $${\left(\frac{\varepsilon_{cc}}{\varepsilon_{co}}\right)}_{con}=2+26.4\left(\frac{f_{ls}}{f_{c}^{\prime }}+{\left(\frac{f_{lf}}{f_{c}^{\prime }}\right)}^{0.7}\right)$$

(13) $$f_{lf}=\frac{2E_{f}n_{f}t_{f}\varepsilon_{fe}}{D}$$

(14) $$f_{ls}=0.5k_{v}\rho_{hs}f_{yh}$$

where *f*_lf_ and *f*_ls_ (MPa) are the lateral confinement pressures provided by the FRP wrap and internal steel reinforcement, respectively; *ɛ_fe_* is the actual rupture strain of the FRP wrap and is considered to be equal to 0.8 times the *ɛ_fu_* value [23].

### 4.5. Analytical Prediction of Slenderness Limit

**Figure 5:**
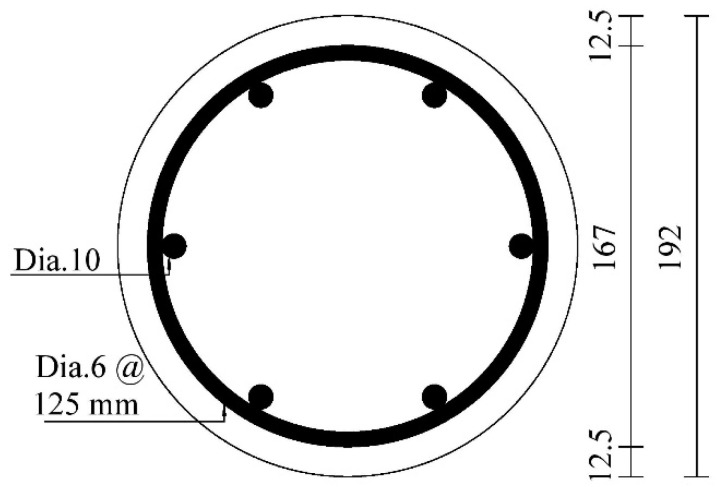
Cross-sectional and steel reinforcement details of specimen selected from [26] for a parametric analysis.

**Figure 6:**
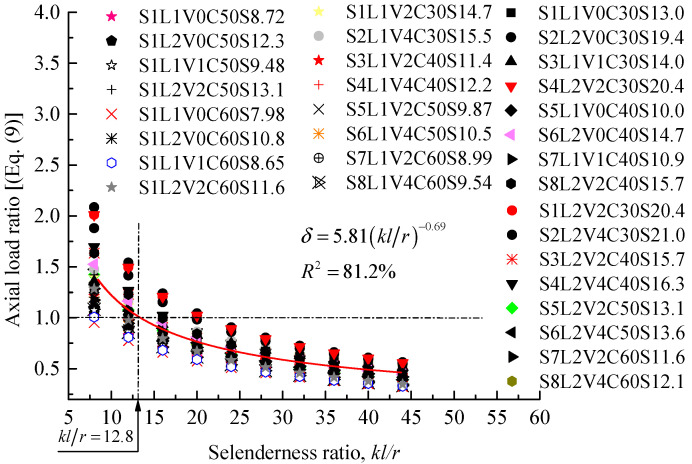
Proposed slenderness limit for FRP-confined concrete columns.

To propose a slenderness limit for FRP-confined RC columns, a total of 32 specimens were designed and analyzed. The control specimen as provided in Figure 5 was selected from [26] for the present parametric study. The amount of longitudinal steel reinforcement and the spacing of the hoop bars were kept the same. The key parameters that are considered were varying amount of hoop and longitudinal FRP (i.e., *n_f_* = 1,2, *n_f,v_* = 0,1,2,4), slenderness ratio (i.e., *kl/r* = 8–44), eccentricity ratio (i.e., *e/D* = 0.1–1.0), and strength of unconfined concrete (i.e., *f_c_^’^* = 30–60 MPa). For example, the symbol S8 in S8L2V4C60S12.1 and its number represent the specimen code of a particular category. The following letter L and its number refer to the number of layers of FRP hoop wraps, whereas V4 refers to the number of layers of FRP longitudinal wraps. The term C60 refers to the concrete type. Finally, the last symbol, S, and the number following it refer to the slenderness ratio. In Figure 6, the results of the proposed model (Equation (9)) are provided, and the regressed formula indicates that the slenderness limit is dependent on the test variables (i.e., FRP confinement ratio), as already confirmed by Pan et al. [62] based on tests on FRP-confined slender RC columns under concentric loading. The slenderness limit is found to be equal to 12.8 (on average). This highlights that designers should apply FRP strengthening in longitudinal direction to ensure that slender CFRP wrapped columns can exhibit improvements in their load-carrying capacity and lateral deformation responses.

**Figure 7:**
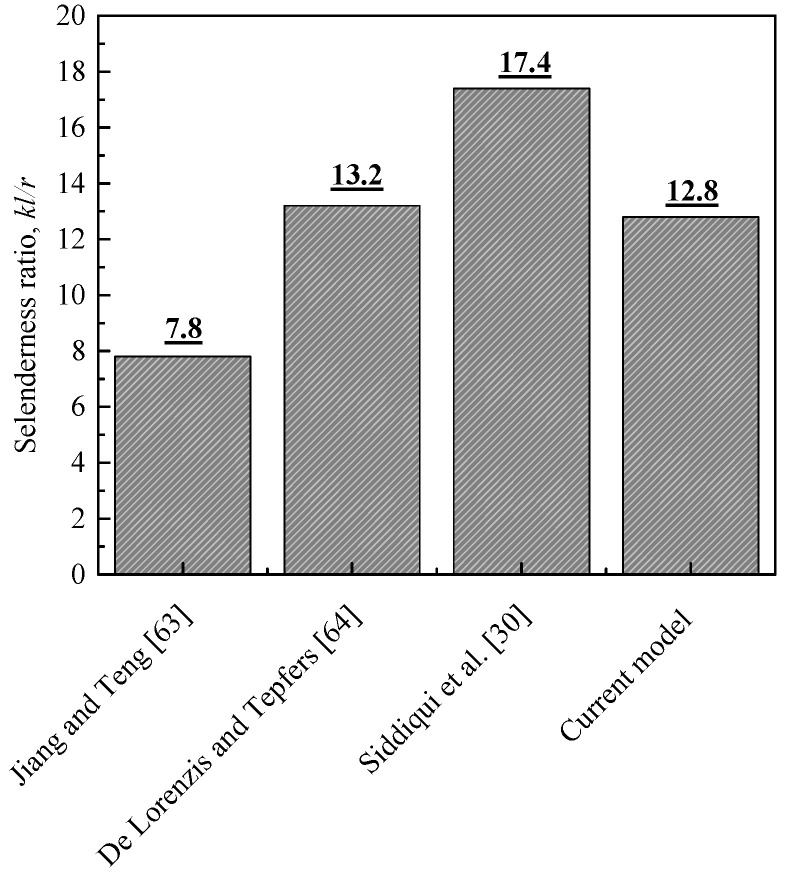
A comparison between models of slenderness limit.

**Figure 8:**
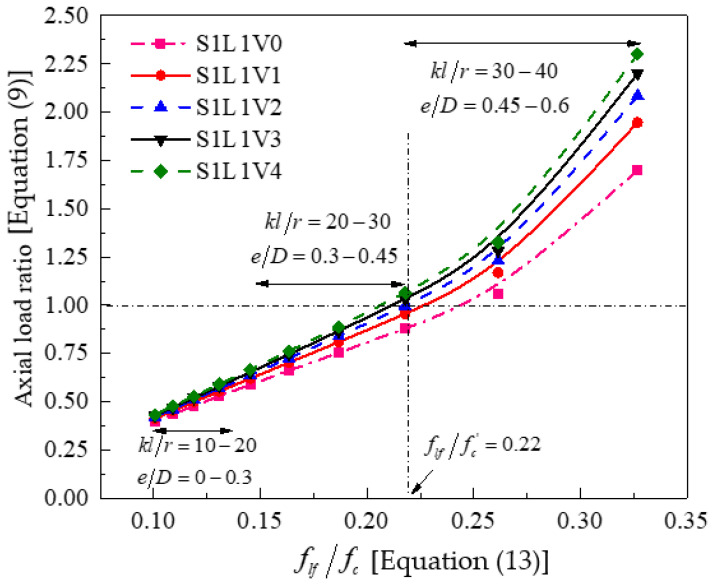
Relationship between effective confinement pressure ratio and confined strength ratio.

Figure 7 compares the slenderness limit proposed by Jiang and Teng [63], De Lorenzis and Tepfers [64], Siddiqui et al. [30], and the present analysis. The chart demonstrates that the slenderness limit values provided by all the investigators, including the present, are less than those of the ACI [65] for the unwrapped RC columns (i.e., *kl/r* = 22). This is attributed to the fact that reductions in strengths of FRP-wrapped columns are higher than those of the unwrapped columns, and that the slenderness effects are more significant for FRP-wrapped columns with higher confinement levels (e.g., [27,28,30]). Generally, it is interesting to report that the averaged result, *kl/r* = 12.8 (see Figure 8), is typical of the averaged result from other models (Figure 7). The satisfactory agreement obtained from these comparisons confirms the accuracy of the present model, and that the effect of the slenderness on column response with different levels of FRP confinement should be accurately estimated.

### 4.6. Minimum Amount of FRP for Adequate Confinement

A confined column needs a minimum amount of FRP wraps for sufficient confinement [66,67,68,69]. In this case, if the axial load *δ*_CRC_ (Equation (9)) is greater than one, the resulting threshold represents the sufficiently confined concrete. Based on an analytical paper by Pham and Hadi [66] on FRP-confined circular and non-circular columns under concentric compression, the minimum limit of effective confinement pressure ratio is proposed to be 0.15.

For columns under eccentric loads, five specimens with different geometry and loading characteristics were studied. The original specimen is similar to that in Figure 5. All specimens had the same steel reinforcement ratio. The analytical variables included longitudinal FRP wraps (i.e., *n_f,v_* = 0,1,2,4), slenderness ratio (i.e., *kl/r* = 10–40), eccentric loading ratio (i.e., *e/D* = 0–0.6), and unconfined concrete strength (i.e., *f*_c_^’^ is from 20 to 65 MPa).

The response between the effective confining pressure ratio and the confined axial load ratio is given in Figure 8. Based on an averaged curve, when *δ*_CRC_ is equal to 1, then the *f_lf_/f_c_^’^* ratio is about 0.22, and such a threshold is larger than that of FRP-confined circular columns under concentric loading due to the reduced effects caused by the eccentric loads. Refer to the discussions of Section 4.2: the results of Figure 8 also confirm that longitudinal FRP sheets for columns under small eccentric ratios are not effective and they can provide greater strength enhancements for slender columns under large eccentricity (e.g., [70,71]).

### 4.7. Complete Stress–Strain Model

According to Ref. [23], A design-oriented stress–strain model for circular unreinforced and reinforced columns strengthened with FRP wraps is presented as follows:

(15) $$y=\frac{Ax+Bx^{2}}{1+Bx+x^{r}}$$

where *x* = *ɛ_c_/ɛ_co_* and *y = f_c_/f_c_^’^*; *ɛ*_c_ and *f_c_* are assumed levels of longitudinal axial strain and stress, respectively. The coefficient *A*, which can be determined from the boundary condition *dσ_c_/dɛ_c_* = *E_c_* at *ɛ_c_* = 0, is provided as follows:

(16) $$A=\frac{E_{c}}{E_{co}}$$

where *E_c_* = 4736$\sqrt{f_{c}^{\prime }}$(MPa) [72] is the elastic modulus of unconfined concrete; *E_co_ = f_c_^’^/ɛ_co_* (MPa) is the secant modulus at the peak stress of unconfined concrete.

(17) $$B=\frac{AX-X^{r}Y-Y}{XY-X^{2}}$$

where *X* = *ɛ_cc_/ɛ_co_* and *Y* = *f_cc_/f_c_^’^*.

**Figure 9:**
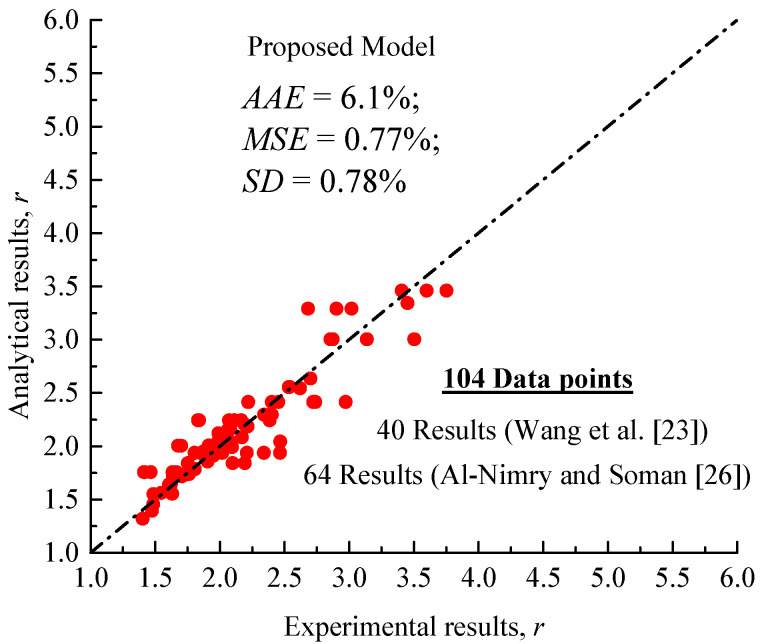
Correlation between experimental and analytical results of the shape factor r estimated using Equation (18).

The parameter *r* in Equation (17) is of significant importance because it controls the overall shape of the stress–strain curve. From two different methodologies of analysis conducted on 64 stress–strain test responses reported by two independent research groups [23,26], the shape factor r can be obtained twice for each curve. This rounded analysis reveals that the factor *r* is related to the lateral confinement provided by the internal steel confinement and external FRP wraps, as well as the contribution made by the longitudinal FRP sheets. Based on these observations, the following model r is proposed and the regressed results are in Figure 9; note that the expressions are calibrated based on specimens of a small range of eccentricity due to the very limited availability of eccentric stress–strain curves of FRP-confined circular RC columns.

(18) $$r=\left\{ \begin{array}{l} \left[B_{6.1}\left(\lambda_{hs}\right)\left(1+\frac{e}{D}\right)+B_{6.2}\left(\lambda_{hs}\right)+B_{6.3}{\left(\lambda_{f}\right)}^{B_{6.4}}+B_{6.5}\left(\lambda_{f,v}\right)\right]{\left(1+\frac{e}{D}\right)}^{B_{6.6}},0\leq \frac{e}{D}\leq 0.26\\ r=B_{7.1}{\left(\lambda_{hs}\right)}^{B_{7.2}}+B_{7.3}{\left(\lambda_{f}\right)}^{B_{7.4}}+B_{7.5}\left(\lambda_{f,v}\right),\frac{e}{D}=0.26 \end{array}\right.$$

where the coefficients *B*_6.1_, *B*_6.2_, *B*_6.3_, *B*_6.4_, *B*_6.5_, *B*_6.6_ in Equation (18) are proposed to be equal to −72.29, 74.16, 1.16, −0.24, 0.41, 2.34, respectively, whereas in its second part *B*_7.1_ = 1.10, *B*_7.2_ = −0.19, *B*_7.3_ = −2.88, *B*_7.4_ = 1.89, *B*_7.5_ = 1.41.

### 4.8. Performance of the Proposed Stress-Strain Model

**Figure 10:**
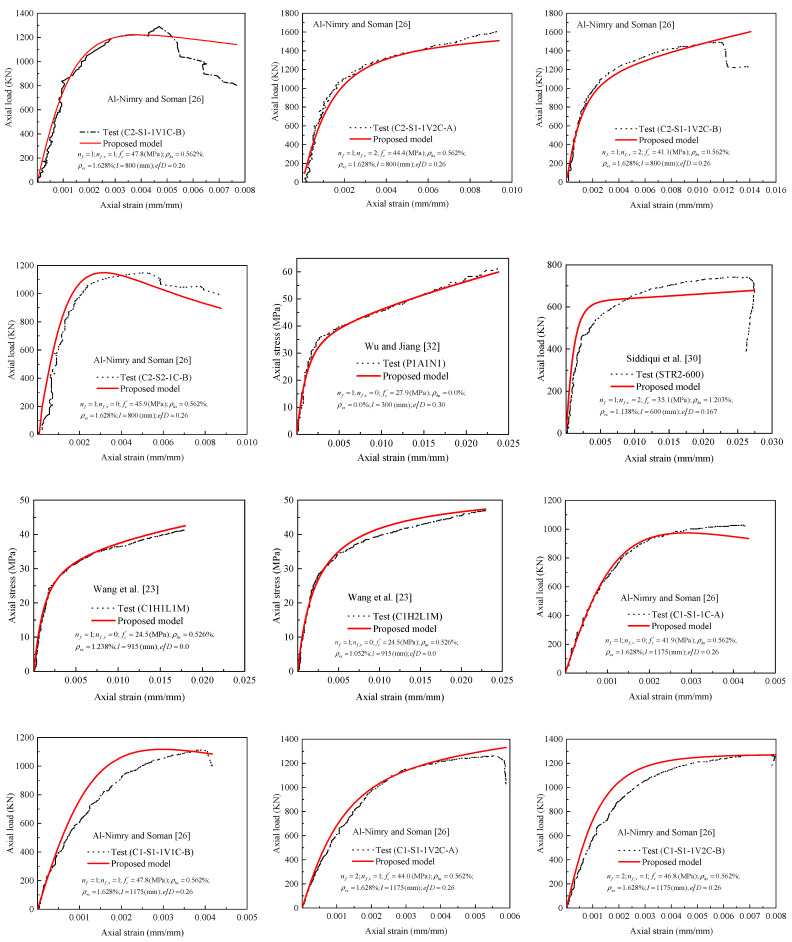

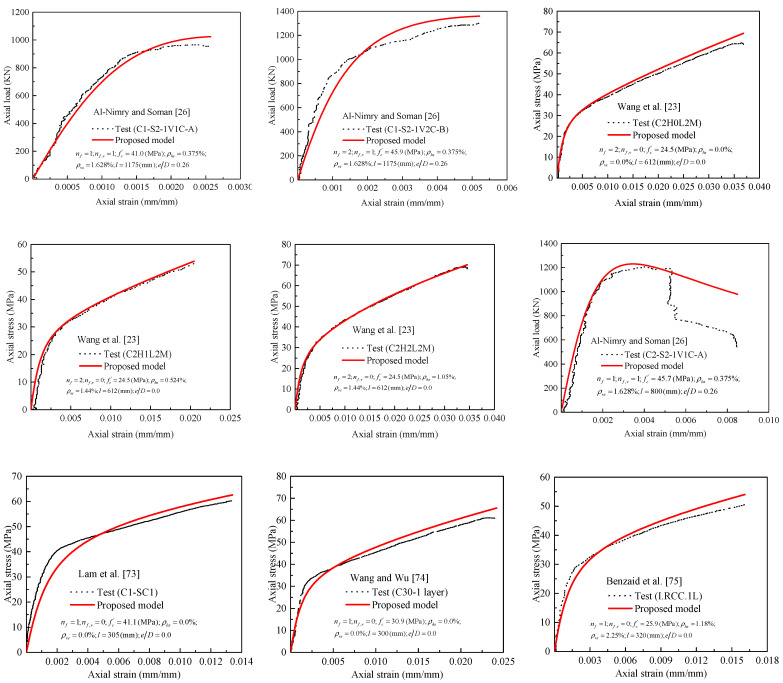
Evaluation of proposed stress–strain model against experimental results.

Figure 10 shows clear comparisons between theoretical stress–strain responses versus tested responses of selected specimens reported in Table A1. The comparisons are from the axial stress and strain data which could be extracted from their original papers. There are no comparisons with results from other tests due to the limited eccentrically loaded responses; however, an additional three concentrically loaded specimens selected from the tests of Lam et al. [73], Wang and Wu [74], and Benzaid et al. [75] to the tests summarized in Table A1 are introduced to calibrate the model. Generally, an inspection of the comparisons demonstrates that the proposed model can capture well the major features of the curve. The shape of stress–strain curves that are well described also reflects the performance and accuracy of the model.

## 5. P–M Interaction Diagrams

### 5.1. Background

**Figure 11:**
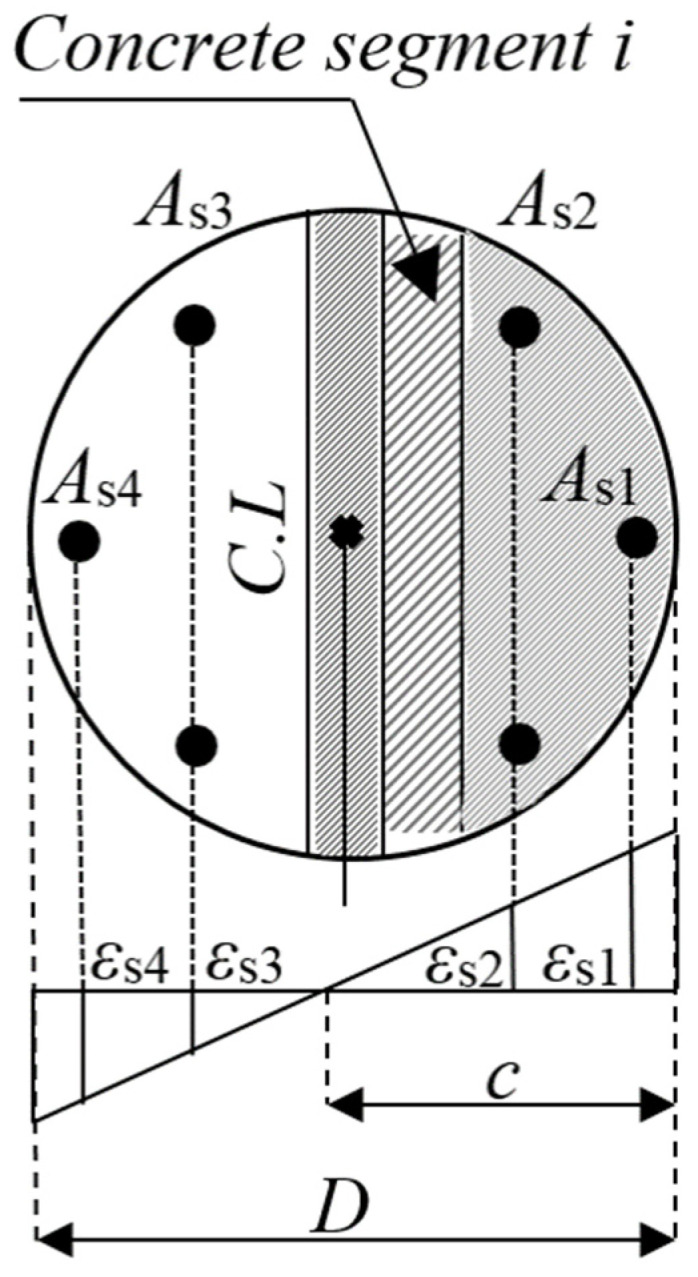
Strain distribution in column cross-section under axial–flexural loading.

Only limited research focusing on the axial load-bending moment response is available for FRP-confined columns (e.g., [25,27,28]). Based on the study provided by Al-Nimry and Al-Rabadi [25], the P–M values of an axial load-bending moment response are calculated using the conventional sectional analysis and considering linear strain variation in the concrete section. While neglecting the contribution of concrete in tension, the concrete in the compression zone is divided into eight equal-width segments (Ref. Figure 11). The concrete strain *ɛ_ci_* at the centroid of *i*th segment is determined using linear trigonometry and the stress *f_ci_* is then calculated using the FRP-confined concrete stress–strain models in Table A2. Assuming a perfect bond between concrete and steel bars, strains in the steel bars were equal to the strains in the adjacent concrete. The tensile and compressive stresses of the steel bars are considered negative and positive in signs, respectively. The force and moment equilibrium expressions are provided as follows:

(19) $$P_{theo}=\sum_{i=1}^{8}A_{ci}f_{ci}+A_{s1}f_{s1}+A_{s2}f_{s2}\pm A_{s3}f_{s3}\pm A_{s4}f_{s4}-A_{v}f_{FRP}$$

(20) $$M_{theo}=\sum_{i=1}^{8}A_{ci}f_{ci}S_{ci}+A_{s1}f_{s1}S_{1}+A_{s2}f_{s2}S_{2}\pm A_{s3}f_{s3}S_{3}\pm A_{s4}f_{s4}S_{4}+A_{v}f_{FRP}z$$

where *A_ci_* is the *i*th concrete segment area; *f_ci_* is the stress at the centroid of the *i*th concrete segment; *A_s_*_1_ to *A_s_*_4_ are section areas of a single reinforcing steel bar (*A_s_*_1_ and *A_s_*_4_ correspond to a single bar, whereas *A_s_*_2_ and *A_s_*_3_ are the areas of 2 reinforcing bars); *f_s_*_1_ to *f_s_*_4_ are the corresponding stress results of the steel bars. The term *S_ci_* is the distance between the column centroid and the center of the segment *I*, and *S*_1_ to *S*_4_ are the distances between the column centroid and the steel reinforcement bars 1 to 4, respectively. The effect of using longitudinal FRP wraps on the column response is also introduced into the above two expressions, in which *A_v_* is the area of longitudinal FRP wraps and is calculated using the geometric properties of a circular segment, *f*_FRP_ is the ultimate tensile strength of FRP wraps, and *z* is the distance between the column’s centroid and the centroid of FRP composites.

### 5.2. Performance of Proposed and Existing P–M Models

**Figure 12:**
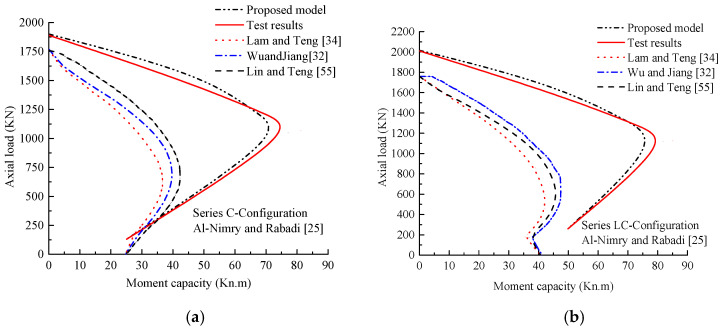
Predicting P–M interaction responses of selected specimens using existing and proposed models. (**a**) Columns strengthened with CFRP sheets only; (**b**) Columns strengthened with both lateral and longitudinal CFRP sheets.

The P–M interaction responses using the newly proposed expressions (Equations (21) and (22)) are shown in Figure 12. The predicted responses obtained using the conventional sectional method in conjunction with the models provided by Lam and Teng [34], Wu and Jiang [32], and Lin and Teng [55] are also provided and assessed. A summary of these models can be found in Table A3. The confined column strength under pure compressive loading was obtained from *N_u_* = 0.85*f_cc_^’^* (*A_g_* − *A_st_*) + *f_y_A_st_*, where the column was considered as unconfined in the case of lower load levels (*N_u_* ≤ 0.1*f_c_^’^A_g_*), and its strength in pure flexure was obtained accordingly. The comparisons included analytical and test P–M responses with different wrapping systems. In Figure 12a, the averaged results of specimens confined with hoop FRP sheets were provided ([25] and Table A1), whereas the averaged results of specimens reinforced with longitudinal and hoop FRP sheets were provided in Figure 12b. The evaluation reveals that the models of FRP-confined unreinforced concrete cylinders have major shortcomings. The predicted results underestimated the tested responses significantly. As noted, before, one reason is the high effectiveness provided by the longitudinal FRP sheets at higher load levels for slender columns when additional moments are developed, and they can greatly enhance the flexural rigidity resistance under combined axial and flexural loads [28]. Generally, the present model exhibits a much better performance in simulating the P–M responses of tested specimens:

(21) $$\Delta =\left[B_{8.1}+B_{8.2}\left(\lambda_{hs}\right)+B_{8.3}{\left(\lambda_{f}\right)}^{B_{8.4}}{\left(\frac{l}{D}\right)}^{B_{8.5}}+B_{8.6}\left(\lambda_{f,v}\right)\right]{\left(\frac{e}{D}\right)}^{B_{8.7}}{\left(\frac{l}{D}\right)}^{B_{8.8}}$$

(22) $$M=N_{u}\left(e+\Delta \right)$$

where the results of parameters *B*_8.1_, *B*_8.2_, *B*_8.3_, *B*_8.4_, *B*_8.5_, *B*_8.6_, *B*_8.7_, *B*_8.8_ in Equation (21) are 0.33, 9.37, 49.21, 0.93, −1.15, −0.31, 0.72, 1.38, respectively.

## 6. Conclusions and Future Research

Based on analytical investigation of a comprehensive database of eccentrically loaded short and slender circular RC columns of varying slenderness ratios and FRP wrapping systems, the following conclusions are drawn as follows:

1. None of the existing design codes and models, among them the GB 50608 [47] and Concrete Society [48], provide accurate predictions for the peak strength and strain, and due to the large test data and parameters studied in the present paper, this finding contradicts a recent conclusion made by Xing et al. [76].
2. The slenderness limit is proposed to be dependent on the FRP confinement level, and the averaged result from the presented model matches well with the averaged results by Jiang and Teng [63], De Lorenzis, and Tepfers [64], and Siddiqui et al. [30].
3. A design-oriented stress–strain model was newly developed using a database of 207 FRP-confined plain and RC columns under different loading conditions. The model parameters included longitudinal and hoop steel reinforcement ratio, amount of FRP hoop wraps, presence of longitudinal FRP sheets, slenderness ratio, eccentric loading ratio, column section’s size, and compressive strength of unconfined concrete.
4. Based on a parametric investigation by the model, the sufficiently confined concrete threshold under eccentric loads was proposed to be 0.22, which is larger than that of Pham and Hadi. [66], since the test database employed in their study mostly contains results of small-scale circular specimens under concentric loading.
5. For slender columns, significantly underestimated predictions of the P–M responses were obtained using both the existing concentric and eccentric stress–strain models of FRP-confined concrete cylinders. However, good agreement between the proposed predictions and tested responses was found, confirming that the model can simulate slender RC columns experiencing greater flexural resistance when strengthened with lateral and longitudinal FRP sheets.

## Data Availability

The data presented in this study are available on request from the corresponding author.

## Appendix A

**Table A1:** Experimental detail of FRP-confined circular specimens.

		Specimens			Concrete	Internal Steel Reinforcement				Fiber-Reinforced Polymer			Key Results			
No.	Specimen	*D* (mm)	*l* (mm)	*e* (mm)	*f*_c_^’^ (MPa)	Hoop reo.	Vertical reo.	*f*_yl_ (MPa)	*f*_yh_ (MPa)	*n_f_*	*n_f,v_*	*t*_f_ (mm)	*f*_f_ (MPa)	*E*_f_ (GPa)	*ε*_fu_ (%)	*N*_u_ (KN)
Al-Nimry and Rahadi [25]																
1	G0-U-A	192	1200	0	58.95	Ø6@96 mm	6Φ10 mm	418	524	0	0	0.166	4900	300	2.1	1529
2	G0-U-B	192	1200	0	58.95	Ø6@96 mm	6Φ10 mm	418	524	0	0	0.166	4900	300	2.1	1573
3	G0-C-A	192	1200	0	58.95	Ø6@96 mm	6Φ10 mm	418	524	1	0	0.166	4900	300	2.1	1930
4	G0-C-B	192	1200	0	58.95	Ø6@96 mm	6Φ10 mm	418	524	1	0	0.166	4900	300	2.1	1872
5	G0-LC-A	192	1200	0	58.95	Ø6@96 mm	6Φ10 mm	418	524	1	1	0.166	4900	300	2.1	1987
6	G0-LC-B	192	1200	0	58.95	Ø6@96 mm	6Φ10 mm	418	524	1	1	0.166	4900	300	2.1	2041
7	G0-LC-C	192	1200	0	58.95	Ø6@96 mm	6Φ10 mm	418	524	1	1	0.166	4900	300	2.1	2021
8	G25-U-A	192	1200	25	58.95	Ø6@96 mm	6Φ10 mm	418	524	0	0	0.166	4900	300	2.1	1351
9	G25-U-B	192	1200	25	58.95	Ø6@96 mm	6Φ10 mm	418	524	0	0	0.166	4900	300	2.1	1154
10	G25-C-A	192	1200	25	58.95	Ø6@96 mm	6Φ10 mm	418	524	1	0	0.166	4900	300	2.1	1545
11	G25-C-B	192	1200	25	58.95	Ø6@96 mm	6Φ10 mm	418	524	1	0	0.166	4900	300	2.1	1580
12	G25-LC	192	1200	25	58.95	Ø6@96 mm	6Φ10 mm	418	524	1	1	0.166	4900	300	2.1	1682
13	G50-U-A	192	1200	50	58.95	Ø6@96 mm	6Φ10 mm	418	524	0	0	0.166	4900	300	2.1	900
14	G50-U-B	192	1200	50	58.95	Ø6@96 mm	6Φ10 mm	418	524	0	0	0.166	4900	300	2.1	894
15	G50-C	192	1200	50	58.95	Ø6@96 mm	6Φ10 mm	418	524	1	0	0.166	4900	300	2.1	1210
16	G50-LC-A	192	1200	50	58.95	Ø6@96 mm	6Φ10 mm	418	524	1	1	0.166	4900	300	2.1	1341
17	G50-LC-B	192	1200	50	58.95	Ø6@96 mm	6Φ10 mm	418	524	1	1	0.166	4900	300	2.1	1303
18	G65-U	192	1200	65	58.95	Ø6@96 mm	6Φ10 mm	418	524	0	0	0.166	4900	300	2.1	789
19	G65-C	192	1200	65	58.95	Ø6@96 mm	6Φ10 mm	418	524	1	0	0.166	4900	300	2.1	1048
20	G65-LC	192	1200	65	58.95	Ø6@96 mm	6Φ10 mm	418	524	1	1	0.166	4900	300	2.1	1122
Al-Nimry and Soman [26]																
21	C1-S1-A	192	1175	50	41.1	Ø6@125 mm	6Φ10 mm	451	528	0	0	0.166	4900	300	2.1	831
22	C1-S1-B	192	1175	50	41.1	Ø6@125 mm	6Φ10 mm	451	528	0	0	0.166	4900	300	2.1	806
23	C1-S1-1C-A	192	1175	50	41.9	Ø6@125 mm	6Φ10 mm	451	528	1	0	0.166	4900	300	2.1	1031
24	C1-S1-1C-B	192	1175	50	41.9	Ø6@125 mm	6Φ10 mm	451	528	1	0	0.166	4900	300	2.1	1043
25	C1-S1-1V1C-A	192	1175	50	44.4	Ø6@125 mm	6Φ10 mm	451	528	1	1	0.166	4900	300	2.1	1011
26	C1-S1-1V1C-B	192	1175	50	47.8	Ø6@125 mm	6Φ10 mm	451	528	1	1	0.166	4900	300	2.1	1122
27	C1-S1-1V-2C-A	192	1175	50	44	Ø6@125 mm	6Φ10 mm	451	528	2	1	0.166	4900	300	2.1	1263
28	C1-S1-1V-2C-B	192	1175	50	46.8	Ø6@125 mm	6Φ10 mm	451	528	2	1	0.166	4900	300	2.1	1273
29	C1-S2-A	192	1175	50	39.5	Ø6@187.5 mm	6Φ10 mm	451	528	0	0	0.166	4900	300	2.1	824
30	C1-S2-B	192	1175	50	39.5	Ø6@187.5 mm	6Φ10 mm	451	528	0	0	0.166	4900	300	2.1	777
31	C1-S2-1C-A	192	1175	50	45.7	Ø6@187.5 mm	6Φ10 mm	451	528	1	0	0.166	4900	300	2.1	941
32	C1-S2-1C-B	192	1175	50	45.7	Ø6@187.5 mm	6Φ10 mm	451	528	1	0	0.166	4900	300	2.1	970
33	C1-S2-1V1C-A	192	1175	50	41	Ø6@187.5 mm	6Φ10 mm	451	528	1	1	0.166	4900	300	2.1	972
34	C1-S2-1V1C-B	192	1175	50	41	Ø6@187.5 mm	6Φ10 mm	451	528	1	1	0.166	4900	300	2.1	946
35	C1-S2-1V-2C-A	192	1175	50	42.7	Ø6@187.5 mm	6Φ10 mm	451	528	2	1	0.166	4900	300	2.1	1290
36	C1-S2-1V-2C-B	192	1175	50	45.9	Ø6@187.5 mm	6Φ10 mm	451	528	2	1	0.166	4900	300	2.1	1302
37	C2-S1-A	192	800	50	44	Ø6@125 mm	6Φ10 mm	451	528	0	0	0.166	4900	300	2.1	879
38	C2-S1-B	192	800	50	44	Ø6@125 mm	6Φ10 mm	451	528	0	0	0.166	4900	300	2.1	867
39	C2-S1-1C-A	192	800	50	46.8	Ø6@125 mm	6Φ10 mm	451	528	1	0	0.166	4900	300	2.1	1205
40	C2-S1-1C-B	192	800	50	46.8	Ø6@125 mm	6Φ10 mm	451	528	1	0	0.166	4900	300	2.1	1309
41	C2-S1-1V1C-A	192	800	50	47.8	Ø6@125 mm	6Φ10 mm	451	528	1	1	0.166	4900	300	2.1	1298
Al-Nimry and Soman [26]																
42	C2-S1-1V1C-B	192	800	50	47.8	Ø6@125 mm	6Φ10 mm	451	528	1	1	0.166	4900	300	2.1	1254
43	C2-S1-1V-2C-A	192	800	50	44.4	Ø6@125 mm	6Φ10 mm	451	528	2	1	0.166	4900	300	2.1	1608
44	C2-S1-1V-2C-B	192	800	50	41.1	Ø6@125 mm	6Φ10 mm	451	528	2	1	0.166	4900	300	2.1	1501
45	C2-S2-A	192	800	50	42.7	Ø6@187.5 mm	6Φ10 mm	451	528	0	0	0.166	4900	300	2.1	815
46	C2-S2-B	192	800	50	42.7	Ø6@187.5 mm	6Φ10 mm	451	528	0	0	0.166	4900	300	2.1	863
47	C2-S2-1C-A	192	800	50	45.9	Ø6@187.5 mm	6Φ10 mm	451	528	1	0	0.166	4900	300	2.1	1210
48	C2-S2-1C-B	192	800	50	45.9	Ø6@187.5 mm	6Φ10 mm	451	528	1	0	0.166	4900	300	2.1	1147
49	C2-S2-1V1C-A	192	800	50	45.7	Ø6@187.5 mm	6Φ10 mm	451	528	1	1	0.166	4900	300	2.1	1214
50	C2-S2-1V1C-B	192	800	50	41	Ø6@187.5 mm	6Φ10 mm	451	528	1	1	0.166	4900	300	2.1	1189
51	C2-S2-1V-2C-A	192	800	50	43.1	Ø6@187.5 mm	6Φ10 mm	451	528	2	1	0.166	4900	300	2.1	1555
52	C2-S2-1V-2C-B	192	800	50	41.1	Ø6@187.5 mm	6Φ10 mm	451	528	2	1	0.166	4900	300	2.1	1405
Bisby and Ranger [27]																
53	U-0	152	608	0	33.2	Ø6.4@100 mm	4Φ6.4 mm	710	710	0	0	0.12	4100	234	1.7	497
54	C-0	152	608	0	33.2	Ø6.4@100 mm	4Φ6.4 mm	710	710	1	0	0.12	4100	234	1.7	873
55	U-5	152	608	5	33.2	Ø6.4@100 mm	4Φ6.4 mm	710	710	0	0	0.12	4100	234	1.7	459
56	C-5	152	608	5	33.2	Ø6.4@100 mm	4Φ6.4 mm	710	710	1	0	0.12	4100	234	1.7	770
57	U-10	152	608	10	33.2	Ø6.4@100 mm	4Φ6.4 mm	710	710	0	0	0.12	4100	234	1.7	447
58	C-10	152	608	10	33.2	Ø6.4@100 mm	4Φ6.4 mm	710	710	1	0	0.12	4100	234	1.7	664
59	U-20	152	608	20	33.2	Ø6.4@100 mm	4Φ6.4 mm	710	710	0	0	0.12	4100	234	1.7	351
60	C-20	152	608	20	33.2	Ø6.4@100 mm	4Φ6.4 mm	710	710	1	0	0.12	4100	234	1.7	579
Fitzwilliam and Bisby [28]																
61	300U-A	152	300	20	30.5	Ø6.4@100 mm	4Φ6.4 mm	693	693	0	0	0.12	4100	234	1.7	467
62	300U-B	152	300	20	30.5	Ø6.4@100 mm	4Φ6.4 mm	693	693	0	0	0.12	4100	234	1.7	460
63	300C-1-0-A	152	300	20	30.5	Ø6.4@100 mm	4Φ6.4 mm	693	693	1	0	0.12	4100	234	1.7	672
64	300C-1-0-B	152	300	20	30.5	Ø6.4@100 mm	4Φ6.4 mm	693	693	1	0	0.12	4100	234	1.7	683
65	300C-1-2-A	152	300	20	30.5	Ø6.4@100 mm	4Φ6.4 mm	693	693	1	2	0.12	4100	234	1.7	681
66	300C-2-0-A	152	300	20	30.5	Ø6.4@100 mm	4Φ6.4 mm	693	693	2	0	0.12	4100	234	1.7	670
67	300C-2-0-B	152	300	20	30.5	Ø6.4@100 mm	4Φ6.4 mm	693	693	2	0	0.12	4100	234	1.7	911
68	600U-A	152	600	20	30.5	Ø6.4@100 mm	4Φ6.4 mm	693	693	0	0	0.12	4100	234	1.7	426
69	600C-1-0-A	152	600	20	30.5	Ø6.4@100 mm	4Φ6.4 mm	693	693	1	0	0.12	4100	234	1.7	561
70	900U-A	152	900	20	30.5	Ø6.4@100 mm	4Φ6.4 mm	693	693	0	0	0.12	4100	234	1.7	397
71	900C-1-0-A	152	900	20	30.5	Ø6.4@100 mm	4Φ6.4 mm	693	693	1	0	0.12	4100	234	1.7	549
72	1200U-A	152	1200	20	30.5	Ø6.4@100 mm	4Φ6.4 mm	693	693	0	0	0.12	4100	234	1.7	388
73	1200U-B	152	1200	20	30.5	Ø6.4@100 mm	4Φ6.4 mm	693	693	0	0	0.12	4100	234	1.7	411
74	1200C-1-0-A	152	1200	20	30.5	Ø6.4@100 mm	4Φ6.4 mm	693	693	1	0	0.12	4100	234	1.7	449
75	1200C-1-0-B	152	1200	20	30.5	Ø6.4@100 mm	4Φ6.4 mm	693	693	1	0	0.12	4100	234	1.7	480
76	1200C-1-2-A	152	1200	20	30.5	Ø6.4@100 mm	4Φ6.4 mm	693	693	1	2	0.12	4100	234	1.7	582
77	1200C-1-4-A	152	1200	20	30.5	Ø6.4@100 mm	4Φ6.4 mm	693	693	1	4	0.12	4100	234	1.7	671
78	1200C-2-0-A	152	1200	20	30.5	Ø6.4@100 mm	4Φ6.4 mm	693	693	2	0	0.12	4100	234	1.7	537
Jiang et al. [29]																
79	L1E0A	150	300	0	38.1	-	-	-	-	1	0	0.11	4743.6	268	1.77	979.5
80	L1E0B	150	300	0	38.1	-	-	-	-	1	0	0.11	4743.6	268	1.77	950.9
81	L1E10A	150	300	10	38.1	-	-	-	-	1	0	0.11	4743.6	268	1.77	779.3
82	L1E10B	150	300	10	38.1	-	-	-	-	1	0	0.11	4743.6	268	1.77	744.8
83	L1E20A	150	300	20	38.1	-	-	-	-	1	0	0.11	4743.6	268	1.77	602.9
84	L1E20B	150	300	20	38.1	-	-	-	-	1	0	0.11	4743.6	268	1.77	610.2
85	L1E30A	150	300	30	38.1	-	-	-	-	1	0	0.11	4743.6	268	1.77	452.1
86	L1E30B	150	300	30	38.1	-	-	-	-	1	0	0.11	4743.6	268	1.77	464.6
87	L2E0A	150	300	0	39.4	-	-	-	-	2	0	0.11	4690.4	266.5	1.76	1306.3
88	L2E0B	150	300	0	39.4	-	-	-	-	2	0	0.11	4690.4	266.5	1.76	1359
89	L2E10A	150	300	10	39.4	-	-	-	-	2	0	0.11	4690.4	266.5	1.76	1099.6
90	L2E10B	150	300	10	39.4	-	-	-	-	2	0	0.11	4690.4	266.5	1.76	1084.6
91	L2E20A	150	300	20	39.4	-	-	-	-	2	0	0.11	4690.4	266.5	1.76	899.6
92	L2E20B	150	300	20	39.4	-	-	-	-	2	0	0.11	4690.4	266.5	1.76	904.4
93	L2E30A	150	300	30	39.4	-	-	-	-	2	0	0.11	4690.4	266.5	1.76	668
94	L2E30B	150	300	30	39.4	-	-	-	-	2	0	0.11	4690.4	266.5	1.76	648.6
Siddiqui et al. [30]																
95	STR1-600	150	600	25	35.1	Ø6@100 mm	4Φ8 mm	420	275	1	0	1	846	77.3	1.1	541.3
96	STR2-600	150	600	25	35.1	Ø6@100 mm	4Φ8 mm	420	275	1	2	1	846	77.3	1.1	745.2
97	STR3-600	150	600	25	35.1	Ø6@100 mm	4Φ8 mm	420	275	1	4	1	846	77.3	1.1	829.9
98	STR2-900	150	900	25	35.1	Ø6@100 mm	4Φ8 mm	420	275	1	2	1	846	77.3	1.1	580.9
99	STR3-900	150	900	25	35.1	Ø6@100 mm	4Φ8 mm	420	275	1	4	1	846	77.3	1.1	660.9
100	STR2-1200	150	1200	25	35.1	Ø6@100 mm	4Φ8 mm	420	275	1	2	1	846	77.3	1.1	545.2
101	STR3-1200	150	1200	25	35.1	Ø6@100 mm	4Φ8 mm	420	275	1	4	1	846	77.3	1.1	647.1
Wang et al. [31]																
102	P-E0-1	150	300	0	37.7	-	-	-	-	0	0	0.167	3400	240	1.6	664
103	P-E0-2	150	300	0	37.7	-	-	-	-	2	0	0.167	3400	240	1.6	1542
104	F-E0-1	150	300	0	37.7	-	-	-	-	2	0	0.167	3400	240	1.6	1612
105	F-E0-2	150	300	0	37.7	-	-	-	-	2	0	0.167	3400	240	1.6	1053
106	F-E15-1	150	300	15	37.7	-	-	-	-	2	0	0.167	3400	240	1.6	1069
107	F-E15-2	150	300	15	37.7	-	-	-	-	2	0	0.167	3400	240	1.6	802
108	F-E25-1	150	300	25	37.7	-	-	-	-	2	0	0.167	3400	240	1.6	790
109	F-E25-2	150	300	25	37.7	-	-	-	-	0	0	0.167	3400	240	1.6	664
Wu and Jiang [32]																
110	A0E0	150	300	0	21.2	-	-	-	-	0	0	0.167	4192	254	1.84	370.8
111	B0E0	150	300	0	21.2	-	-	-	-	0	0	0.167	4192	254	1.84	379.8
112	A0E10	150	300	10	21.2	-	-	-	-	0	0	0.167	4192	254	1.84	337.8
113	B0E10	150	300	10	21.2	-	-	-	-	0	0	0.167	4192	254	1.84	330.9
114	A0E20	150	300	20	21.2	-	-	-	-	0	0	0.167	4192	254	1.84	300.9
115	B0E20	150	300	20	21.2	-	-	-	-	0	0	0.167	4192	254	1.84	291.9
116	A0E30	150	300	30	21.2	-	-	-	-	0	0	0.167	4192	254	1.84	288.9
117	B0E30	150	300	30	21.2	-	-	-	-	0	0	0.167	4192	254	1.84	271.9
Wu and Jiang [32]																
118	A0E40	150	300	40	21.2	-	-	-	-	0	0	0.167	4192	254	1.84	248.9
119	B0E40	150	300	40	21.2	-	-	-	-	0	0	0.167	4192	254	1.84	238.9
120	A0E50	150	300	50	21.2	-	-	-	-	0	0	0.167	4192	254	1.84	202.9
121	B0E50	150	300	50	21.2	-	-	-	-	0	0	0.167	4192	254	1.84	185.9
122	A1E0	150	300	0	28.7	-	-	-	-	1	0	0.167	4192	254	1.84	1048.6
123	B1E0	150	300	0	28.7	-	-	-	-	1	0	0.167	4192	254	1.84	968.7
124	A1E10	150	300	10	28.7	-	-	-	-	1	0	0.167	4192	254	1.84	938.7
125	B1E10	150	300	10	28.7	-	-	-	-	1	0	0.167	4192	254	1.84	880.7
126	A1E20	150	300	20	28.7	-	-	-	-	1	0	0.167	4192	254	1.84	850.7
127	B1E20	150	300	20	28.7	-	-	-	-	1	0	0.167	4192	254	1.84	739.7
128	A1E30	150	300	30	28.7	-	-	-	-	1	0	0.167	4192	254	1.84	755.7
129	B1E30	150	300	30	28.7	-	-	-	-	1	0	0.167	4192	254	1.84	768.7
130	A1E40	150	300	40	28.7	-	-	-	-	1	0	0.167	4192	254	1.84	691.7
131	B1E40	150	300	40	28.7	-	-	-	-	1	0	0.167	4192	254	1.84	633.8
132	A1E50	150	300	50	28.7	-	-	-	-	1	0	0.167	4192	254	1.84	474.8
133	B1E50	150	300	50	28.7	-	-	-	-	1	0	0.167	4192	254	1.84	434.8
134	A2E0	150	300	0	30.1	-	-	-	-	2	0	0.167	4192	254	1.84	1557.5
Wu and Jiang [32]																
135	B2E0	150	300	0	30.1	-	-	-	-	2	0	0.167	4192	254	1.84	1597.5
136	A2E10	150	300	10	30.1	-	-	-	-	2	0	0.167	4192	254	1.84	1463.5
137	B2E10	150	300	10	30.1	-	-	-	-	2	0	0.167	4192	254	1.84	1434.5
138	A2E20	150	300	20	30.1	-	-	-	-	2	0	0.167	4192	254	1.84	1267.6
139	B2E20	150	300	20	30.1	-	-	-	-	2	0	0.167	4192	254	1.84	1349.5
140	A2E30	150	300	30	30.1	-	-	-	-	2	0	0.167	4192	254	1.84	1164.6
141	B2E30	150	300	30	30.1	-	-	-	-	2	0	0.167	4192	254	1.84	1201.6
142	A2E40	150	300	40	30.1	-	-	-	-	2	0	0.167	4192	254	1.84	908.7
143	B2E40	150	300	40	30.1	-	-	-	-	2	0	0.167	4192	254	1.84	862.7
144	A2E50	150	300	50	30.1	-	-	-	-	2	0	0.167	4192	254	1.84	704.7
145	B2E50	150	300	50	30.1	-	-	-	-	2	0	0.167	4192	254	1.84	678.7
Wang et al. [23]																
146	C1H1L0M	305	915	0	24.5	Ø6@80 mm	8Φ12 mm	340	397	0	-	0.167	4340	244	1.78	41.5
147	C1H2L0M	305	915	0	24.5	Ø6@40 mm	8Φ12 mm	340	397	0	-	0.167	4340	244	1.78	62.1
148	C1H1L1M	305	915	0	24.5	Ø6@80 mm	8Φ12 mm	340	397	1	-	0.167	4340	244	1.78	41.5
149	C1H1L1C	305	915	0	24.5	Ø6@80 mm	8Φ12 mm	340	397	1	-	0.167	4340	244	1.78	43.1
150	C1H0L1M	305	915	0	24.5	-	-	-	397	1	-	0.167	4340	244	1.78	35.0
151	C1H0L2M	305	915	0	24.5	-	-	-	397	2	-	0.167	4340	244	1.78	55.3
152	C1H1L2M	305	915	0	24.5	Ø6@80 mm	8Φ12 mm	340	397	2	-	0.167	4340	244	1.78	52.2
153	C1H1L2C	305	915	0	24.5	Ø6@80 mm	8Φ12 mm	340	397	2	-	0.167	4340	244	1.78	61.8
154	C1H2L1M	305	915	0	24.5	Ø6@40 mm	8Φ12 mm	340	397	1	-	0.167	4340	244	1.78	47.0
155	C1H2L2M	305	915	0	24.5	Ø6@40 mm	8Φ12 mm	340	397	2	-	0.167	4340	244	1.78	62.1
156	C2H1L0M	204	612	0	24.5	Ø6@120 mm	6Φ10 mm	312	397	0	-	0.167	4340	244	1.78	52.1
157	C2H2L0M	204	612	0	24.5	Ø6@60 mm	6Φ10 mm	312	397	0	-	0.167	4340	244	1.78	52.2
158	C2H1L1M	204	612	0	24.5	Ø6@120 mm	6Φ10 mm	312	397	1	-	0.167	4340	244	1.78	52.1
159	C2H1L1C	204	612	0	24.5	Ø6@120 mm	6Φ10 mm	312	397	1	-	0.167	4340	244	1.78	49.9
160	C2H1L2M	204	612	0	24.5	Ø6@120 mm	6Φ10 mm	312	397	2	-	0.167	4340	244	1.78	66.1
161	C2H1L2C	204	612	0	24.5	Ø6@120 mm	6Φ10 mm	312	397	2	-	0.167	4340	244	1.78	68.9
162	C2H0L1M	204	612	0	24.5	-	-	-	397	1	-	0.167	4340	244	1.78	46.1
163	C2H0L1C	204	612	0	24.5	-	-	-	397	1	-	0.167	4340	244	1.78	42.3
164	C2H0L2M	204	612	0	24.5	-	-	-	397	2	-	0.167	4340	244	1.78	65.2
165	C2H0L2C	204	612	0	24.5	-	-	-	397	2	-	0.167	4340	244	1.78	66.8
166	C2H2L1M	204	612	0	24.5	Ø6@60 mm	6Φ10 mm	312	397	1	-	0.167	4340	244	1.78	52.2
167	C2H2L1C	204	612	0	24.5	Ø6@60 mm	6Φ10 mm	312	397	1	-	0.167	4340	244	1.78	57.0
168	C2H2L2M	204	612	0	24.5	Ø6@60 mm	6Φ10 mm	312	397	2	-	0.167	4340	244	1.78	69.5
169	C2H2L2C	204	612	0	24.5	Ø6@60 mm	6Φ10 mm	312	397	2	-	0.167	4340	244	1.78	75.0
Kaeseberg et al. [57]																
170	D15-TR-M1-2L-1	150	300	0	42.3	Ø6@100 mm	6Φ8 mm	550	550	2	0	0.111	3900	230	1.70	83.8
171	D15-TR-M1-2L-1	150	300	0	42.3	Ø6@100 mm	6Φ8 mm	550	550	2	0	0.111	3900	230	1.70	89.5
172	D15-TR-M1-2L-1	150	300	0	42.3	Ø6@100 mm	6Φ8 mm	550	550	2	0	0.111	3900	230	1.70	86.2
173	D15-TR-M1-2L-2	150	300	0	42.3	Ø6@50 mm	6Φ8 mm	550	550	2	0	0.111	3900	230	1.70	83.3
174	D15-TR-M1-2L-2	150	300	0	42.3	Ø6@50 mm	6Φ8 mm	550	550	2	0	0.111	3900	230	1.70	81.9
175	D15-TR-M1-2L-2	150	300	0	42.3	Ø6@50 mm	6Φ8 mm	550	550	2	0	0.111	3900	230	1.70	73.0
176	D20-TR-M1-2L-1	200	400	0	27.0	Ø4@175 mm	6Φ12 mm	500	550	2	0	0.111	3900	230	1.70	65.1
177	D20-TR-M1-2L-1	200	400	0	27.0	Ø4@175 mm	6Φ12 mm	500	550	2	0	0.111	3900	230	1.70	69.4
178	D20-TR-M1-2L-1	200	400	0	27.0	Ø4@175 mm	6Φ12 mm	500	550	2	0	0.111	3900	230	1.70	67.8
179	D20-TR-M1-2L-2	200	400	0	27.0	Ø6@175 mm	6Φ12 mm	500	550	2	0	0.111	3900	230	1.70	65.0
180	D20-TR-M1-2L-2	200	400	0	27.0	Ø6@175 mm	6Φ12 mm	500	550	2	0	0.111	3900	230	1.70	64.4
181	D20-TR-M1-2L-2	200	400	0	27.0	Ø6@175 mm	6Φ12 mm	500	550	2	0	0.111	3900	230	1.70	60.8
182	D20-TR-M2-2L-3a	200	400	0	28.0	Ø6@100 mm	4Φ12 mm	500	550	2	0	0.111	4100	230	1.78	66.1
183	D20-TR-M2-2L-3a	200	400	0	28.0	Ø6@100 mm	4Φ12 mm	500	550	2	0	0.111	4100	230	1.78	68.7
184	D20-TR-M2-2L-3a	200	400	0	28.0	Ø6@100 mm	4Φ12 mm	500	550	2	0	0.111	4100	230	1.78	67.1
185	D20-TR-M2-2L-3b	200	400	0	28.0	Ø6@100 mm	6Φ12 mm	500	550	2	0	0.111	4100	230	1.78	72.8
186	D20-TR-M2-2L-3b	200	400	0	28.0	Ø6@100 mm	6Φ12 mm	500	550	2	0	0.111	4100	230	1.78	75.9
187	D20-TR-M2-2L-3b	200	400	0	28.0	Ø6@100 mm	6Φ12 mm	500	550	2	0	0.111	4100	230	1.78	72.8
188	D20-TR-M2-2L-3c	200	400	0	28.0	Ø6@100 mm	8Φ12 mm	500	550	2	0	0.111	4100	230	1.78	76.3
189	D20-TR-M2-2L-3c	200	400	0	28.0	Ø6@100 mm	8Φ12 mm	500	550	2	0	0.111	4100	230	1.78	77.1
190	D20-TR-M2-2L-3c	200	400	0	28.0	Ø6@100 mm	8Φ12 mm	500	550	2	0	0.111	4100	230	1.78	78.4
191	D20-TR-M2-2L-4	200	400	0	28.0	Ø6@50 mm	6Φ12 mm	500	550	2	0	0.111	4100	230	1.78	77.0
192	D20-TR-M2-2L-4	200	400	0	28.0	Ø6@50 mm	6Φ12 mm	500	550	2	0	0.111	4100	230	1.78	77.1
193	D20-TR-M2-2L-4	200	400	0	28.0	Ø6@50 mm	6Φ12 mm	500	550	2	0	0.111	4100	230	1.78	78.1
194	D20-TR-M2-1L-1	200	400	0	24.5	Ø6@75 mm	6Φ12 mm	500	550	1	0	0.111	4100	230	1.78	51.6
195	D20-TR-M2-1L-1	200	400	0	24.5	Ø6@75 mm	6Φ12 mm	500	550	1	0	0.111	4100	230	1.78	54.3
196	D20-TR-M2-1L-2	200	400	0	24.5	Ø6@75 mm	6Φ12 mm	500	730	1	0	0.111	4100	230	1.78	49.1
197	D20-TR-M2-1L-2	200	400	0	24.5	Ø6@75 mm	6Φ12 mm	500	730	1	0	0.111	4100	230	1.78	57.0
198	D20-TR-M2-1L-2	200	400	0	24.5	Ø6@75 mm	6Φ12 mm	500	730	1	0	0.111	4100	230	1.78	56.7
199	D20-TR-M2-1L-3	200	400	0	24.5	Ø5@50 mm	6Φ12 mm	500	670	1	0	0.111	4100	230	1.78	56.7
200	D20-TR-M2-1L-3	200	400	0	24.5	Ø5@50 mm	6Φ12 mm	500	670	1	0	0.111	4100	230	1.78	57.8
201	D20-TR-M2-1L-3	200	400	0	24.5	Ø5@50 mm	6Φ12 mm	500	670	1	0	0.111	4100	230	1.78	52.1
202	D25-TR-M1-2L-1	250	500	0	33.0	Ø6@100 mm	6Φ12 mm	500	550	2	0	0.111	3900	230	1.70	60.9
203	D25-TR-M1-2L-1	250	500	0	33.0	Ø6@100 mm	6Φ12 mm	500	550	2	0	0.111	3900	230	1.70	57.6
204	D25-TR-M1-2L-1	250	500	0	33.0	Ø6@100 mm	6Φ12 mm	500	550	2	0	0.111	3900	230	1.70	50.8
205	D25-TR-M1-2L-2	250	1000	0	31.2	Ø6@100 mm	6Φ12 mm	500	550	2	0	0.111	3900	230	1.70	54.0
206	D25-TR-M1-2L-2	250	1000	0	31.2	Ø6@100 mm	6Φ12 mm	500	550	2	0	0.111	3900	230	1.70	50.8
207	D25-TR-M1-2L-2	250	1000	0	31.2	Ø6@100 mm	6Φ12 mm	500	550	2	0	0.111	3900	230	1.70	54.6

**Table A2:** Summary of existing confined stress and corresponding strain models.

	For Confined Stress	For Confined Strain	Model Parameters
GB 50608 (GB2010) [47]	$f_{cc,con}^{\prime }=f_{c}^{\prime }+3.5\frac{E_{f}n_{f}t_{f}}{R}\left(1-\frac{6.5}{\beta_{j}}\right)\varepsilon_{fe}$	$\varepsilon_{cc,con}=0.0033+0.6\beta_{j}{}^{0.8}\varepsilon_{fe}{}^{1.45}$	$\beta_{j}=\frac{E_{f}n_{f}t_{f}}{f_{c}^{\prime }R}$
Concrete Society [48]	$\frac{f_{cc,con}^{\prime }}{f_{co}^{\prime }}=1+5.25\left(\rho_{K}-0.01\right)\rho_{\varepsilon }$	$\frac{\varepsilon_{cc,con}}{\varepsilon_{co}}=1.75+6.5\rho_{K}{}^{0.8}\rho_{\varepsilon }{}^{1.45}$	$\rho_{K}=\frac{2E_{f}t_{f}}{D\left(f_{c}^{\prime }/\varepsilon_{co}\right)}\geq 0.01;\rho_{\varepsilon }=\varepsilon_{fe}/\varepsilon_{co};f_{co}^{\prime }=0.85f_{c}^{\prime }$
El Maaddawy [39]	$f_{cc,ecc}^{\prime }=f_{c}^{\prime }+\left(\left(f_{c}^{\prime }\left(1+2\frac{f_{lfe}}{f_{c}^{\prime }}\right)\right)-f_{c}^{\prime }\right)\left(\frac{1}{1+e/h}\right)$	$\varepsilon_{cc,ecc}=\varepsilon_{cu}+\left(\left(\varepsilon_{co}\left(1.75+10\frac{f_{lfe}}{f_{c}^{\prime }}\right)\right)-\varepsilon_{cu}\right)\left(\frac{1}{1+e/h}\right)$	$f_{lfe}=k_{s}\frac{2f_{f}t_{f}n_{f}}{\frac{1}{2}(b+h)};t_{f}=\left\{ \begin{array}{l} t_{f}(Full-wrapping)\\ \frac{w_{f}}{s_{f}}t_{f}(Partial-wrapping) \end{array}\right.$$\kappa_{s}=\left[1-\frac{{\left(b-2r_{c}\right)}^{2}+{\left(h-2r_{c}\right)}^{2}}{3A_{c}}\right]/\rho_{vs}$ $\kappa_{s}$ is given by Sheikh and Uzumeri [77], Chaallal and Shahawy [78], Teng et al. [79].
Hu et al. [41]	$\frac{f_{cc,con}^{\prime }}{f_{c}^{\prime }}=1+2.0\kappa_{s}^{2.50}\frac{f_{lf}}{f_{c}^{\prime }}$ (Hu and Wang [80]) $\frac{f_{cc,ecc}^{\prime }}{f_{c}^{\prime }}=\frac{f_{cc,con}^{\prime }}{f_{c}^{\prime }}$	$\frac{\varepsilon_{cc,con}}{\varepsilon_{co}}=1+26.2\kappa_{s}^{0.12}{\left(\frac{f_{lf}}{f_{c}^{\prime }}\right)}^{0.80}E_{l}^{-0.148}$ (Hu and Wang [80]) $\varepsilon_{cc,ecc}=1.53\varepsilon_{cc}{\left(1+\frac{e}{h}\right)}^{-0.37}{\left(\frac{l}{D}\right)}^{-0.35}$	$\begin{array}{l} f_{lf}=\frac{2n_{f}t_{f}f_{f}}{b}\\ E_{l}=\frac{2n_{f}t_{f}E_{f}}{b} \end{array}$ $\kappa_{s}=1-\frac{\left(b/h\right){\left(h-2r_{c}\right)}^{2}+\left(h/b\right){\left(b-2r_{c}\right)}^{2}}{3\left[bh-\left(4-\pi \right)r_{c}{}^{2}\right]\left(1-\rho_{vs}\right)}$ (Lam and Teng [81])

Note: The definitions of the model coefficients are provided in Table A4.

**Table A3:** Summary of existing stress–strain models.

	For Stress–Strain Response	For Confined Stress and Strain
Lam and Teng [34]	$f_{c}=\left\{ \begin{array}{l} E_{c}\varepsilon_{c}-\frac{{\left(E_{c}-E_{2}\right)}^{2}}{4f_{c}^{\prime }},0\leq \varepsilon_{c}\leq \varepsilon_{t}\\ f_{c}^{\prime }+E_{2,con}\varepsilon_{c},\varepsilon_{t}\leq \varepsilon_{c}\leq \varepsilon_{cc} \end{array}\right.;E_{2,con}=\frac{f_{cc}^{\prime }-f_{c}^{\prime }}{\varepsilon_{cc}};\varepsilon_{t}=\frac{2f_{c}^{\prime }}{E_{c}-E_{2,con}}$ (ACI, 440.2R-17 [46])	$f_{cc,con}^{\prime }=f_{c}^{\prime }+\psi_{f}3.3f_{lf};\psi_{f}=0.95;f_{lf}=\frac{2E_{f}n_{f}t_{f}\varepsilon_{fe}}{D};\varepsilon_{fe}=\kappa_{\varepsilon }\varepsilon_{fu};$ $\kappa_{\varepsilon }=0.55$ (ACI, 440.2R-17 [46]) $\varepsilon_{cc,con}=\varepsilon_{co}\left(1.5+12\kappa_{s}\frac{f_{lf}}{f_{c}^{\prime }}{\left(\frac{\varepsilon_{fe}}{\varepsilon_{co}}\right)}^{0.45}\right)\leq 0.01;$ $\varepsilon_{co}=0.000937{\left(f_{c}^{\prime }\right)}^{0.25}$ (Popovics [82])
Wu and Jiang [32]	$\begin{array}{l} f_{c}=\left[\left(E_{1}\varepsilon_{n}-f_{o,ecc}\right)e^{-\varepsilon_{c}/\varepsilon_{n}}+f_{o,ecc}+E_{2,ecc}\varepsilon_{c}\right]\left(1-e^{-\varepsilon_{c}/\varepsilon_{n}}\right);\frac{E_{2,ecc}}{E_{2,con}}=1+\left[5.55{\left(\frac{e}{R}\right)}^{2.49}{\left(\frac{E_{f}t_{f}}{E_{1}R}\right)}^{0.11}\right]k\\ k=1.3-0.84{\left(\frac{e}{R}\right)}^{1.74}{\left(\frac{N}{A_{c}f_{c}^{\prime }}\right)}^{1.1};\frac{f_{o,ecc}}{f_{o,con}}=1+7.02{\left(\frac{e}{R}\right)}^{1.67}{\left(\frac{E_{f}t_{f}}{E_{1}R}\right)}^{0.32};\varepsilon_{n}=n\varepsilon_{o,ecc};\varepsilon_{o}=f_{o,ecc}/E_{1} \end{array}$	-
Lin and Teng [55]	$\begin{array}{l} f_{c}=\left\{ \begin{array}{l} E_{c}\varepsilon_{c}-\frac{{\left(E_{c}-E_{2,ecc}\right)}^{2}}{4f_{c}^{\prime }}\varepsilon_{c}^{2},0\leq \varepsilon_{c}<\varepsilon_{t}\;\;\;\;\\ f_{c}^{\prime }+E_{2,ecc}\varepsilon_{c},\varepsilon_{t}\leq \varepsilon_{c}\leq \varepsilon_{ccu,ecc} \end{array}\right.(E_{2,ecc}\geq 0);f_{c}=\left\{ \begin{array}{l} E_{c}\varepsilon_{c}-\frac{E_{c}^{2}}{4f_{c}^{\prime }}\varepsilon_{c}^{2},0\leq \varepsilon_{c}<\varepsilon_{t}\\ f_{t}+E_{2,ecc}\left(\varepsilon_{c}-\varepsilon_{t}\right),\varepsilon_{t}\leq \varepsilon_{c}\leq \varepsilon_{ccu,ecc} \end{array}\right.(E_{2,ecc}<0)\\ \varepsilon_{t}=\left\{ \begin{array}{l} \frac{2f_{c}^{\prime }}{E_{c}-E_{2,ecc}},(E_{2,ecc}\geq 0)\\ \frac{2f_{c}^{\prime }\left(E_{c}-E_{2,ecc}\right)}{E_{c}^{2}},(E_{2,ecc}<0) \end{array}\right.;f_{t}=\left\{ \begin{array}{l} f_{c}^{\prime }+E_{2,ecc}\varepsilon_{t},(E_{2,ecc}\geq 0)\\ E_{c}\varepsilon_{t}-\frac{E_{c}^{2}}{4f_{c}^{\prime }}\varepsilon_{t}^{2},(E_{2,ecc}<0) \end{array}\right.\\ E_{2,ecc}=E_{2,con}\left(1-0.0808\frac{E_{2,con}}{\left|E_{2,con}\right|}\frac{D}{c}\right),\frac{D}{c}\leq 12.4 \end{array}$	$\varepsilon_{cc,ecc}=\varepsilon_{cc,con}\left(1+0.263\frac{D}{c}+0.0227{\left(\frac{D}{c}\right)}^{2}\right)$

Note: The definition of the model coefficients are provided in Table A4.

**Table A4:** List of notations.

Notation	Definition	Notation	Definition
*f’_co_* (MPa)	equals to 0.85 times the compressive strength of unconfined concrete	*f_f_* (MPa)	Stress in FRP wraps
*R* (mm)	radius of a column cross-section	*A_c_* (mm^2^)	cross-sectional area of concrete column
*E_f_* (MPa)	elastic modulus of the FRP in the lateral direction	*r_c_* (mm)	the radius of rounded rectangular column section
*t_f_* (mm)	nominal thickness of a single FRP sheet	*ρ_vs_*	Longitudinal steel reinforcement ratio
*n_f_*	total number of FRP wraps in the lateral direction	*w_f_* (mm)	width of partially wrapped FRP sheet
*ɛ**_fe_* (mm/mm)	the actual strain of FRP hoop wraps at rupture	*s_f_* (mm)	center to center spacing between partial wrapping sheets
*β_j,_* *ρ* *_k_*	lateral FRP confinement stiffness	*K_s_*	shape factor to account for a rectangular rounded section (equals to 1 for circular sectioned columns)
*ρ* *_ɛ_*	strain ratio	*ɛ**_cu_* (mm/mm)	assumed to be 0.004 (Park and Paulay [83])
*f’_cc,con_* (MPa)	strength of FRP-confined concrete under pure compression	*E_l_* (MPa)	lateral confining modulus of FRP wraps
*ɛ**_cc,con_* (mm/mm)	strain of FRP-confined concrete under pure compression	*f_c_* (MPa)	axial stress on a stress–strain curve
*f_lf_* (MPa)	lateral confining pressure provided by the FRP wraps	*ɛ**_c_* (mm/mm)	the corresponding axial strain on a stress–strain curve
*f_lfe_* (MPa)	effective lateral confining pressure provided by the FRP wraps	*E_c_* (MPa)	elastic modulus of unconfined concrete
*e* (mm)	load eccentricity	*E_2,con_* (MPa)	the slope of the second branch of a pure compression stress–strain curve
*b* (mm)	width of column section	*ψ_f_*	taken to be equal to 0.95 (Lam and Teng [34])
*h* (mm)	depth of column section	*ɛ**_co_* (mm/mm)	maximum strain of unconfined concrete
*K* *_ɛ _*	the strain efficiency factor of FRP wraps	*f_o,ecc_* (MPa)	the stress coordinate of the intersection between the line along the second branch of a stress–strain curve and the line parallel to the first parabolic branch
*n*	the curve shape parameter that describes the transition zone	*E_1_* (MPa)	is considered to be equal to *E_c_* (MPa) (e.g., Wu and Jiang [32])
*ɛ**_t_* (mm/mm)	transition strain between the first and second parts of the stress–strain curve	*f_t_* (MPa)	transition stress between the first and second parts of the stress–strain curve
*ɛ**_cc,ecc_* (mm/mm)	similar to *ɛ**_cc,con_* but for concentric loading	*D/c*	diameter of column section to the depth of compression zone
*E_2,ecc_* (MPa)	the slope of the second branch of an eccentric stress–strain curve	*f’_cc,ecc_* (MPa)	similar to *f’_cc,con_* but for concentric loading
*ɛ**_cc,ecc_* (mm/mm)	the strain of FRP-confined concrete under eccentric compression	*f_o,con_* (MPa)	similar to *f_o,con_* but for concentric loading
*D* (mm)	diameter of a circularcolumn section	*n_f,v_*	total number of FRP wraps in the longitudinal direction
*l* (mm)	column height	*N_u_* (KN)	maximum load capacity
